# Association of breast cancer risk in *BRCA1* and *BRCA2* mutation carriers with genetic variants showing differential allelic expression: identification of a modifier of breast cancer risk at locus 11q22.3

**DOI:** 10.1007/s10549-016-4018-2

**Published:** 2016-10-28

**Authors:** Yosr Hamdi, Penny Soucy, Karoline B. Kuchenbaeker, Tomi Pastinen, Arnaud Droit, Audrey Lemaçon, Julian Adlard, Kristiina Aittomäki, Irene L. Andrulis, Adalgeir Arason, Norbert Arnold, Banu K. Arun, Jacopo Azzollini, Anita Bane, Laure Barjhoux, Daniel Barrowdale, Javier Benitez, Pascaline Berthet, Marinus J. Blok, Kristie Bobolis, Valérie Bonadona, Bernardo Bonanni, Angela R. Bradbury, Carole Brewer, Bruno Buecher, Saundra S. Buys, Maria A. Caligo, Jocelyne Chiquette, Wendy K. Chung, Kathleen B. M. Claes, Mary B. Daly, Francesca Damiola, Rosemarie Davidson, Miguel De la Hoya, Kim De Leeneer, Orland Diez, Yuan Chun Ding, Riccardo Dolcetti, Susan M. Domchek, Cecilia M. Dorfling, Diana Eccles, Ros Eeles, Zakaria Einbeigi, Bent Ejlertsen, EMBRACE, Christoph Engel, D. Gareth Evans, Lidia Feliubadalo, Lenka Foretova, Florentia Fostira, William D. Foulkes, George Fountzilas, Eitan Friedman, Debra Frost, Pamela Ganschow, Patricia A. Ganz, Judy Garber, Simon A. Gayther, GEMO Study Collaborators, Anne-Marie Gerdes, Gord Glendon, Andrew K. Godwin, David E. Goldgar, Mark H. Greene, Jacek Gronwald, Eric Hahnen, Ute Hamann, Thomas V. O. Hansen, Steven Hart, John L. Hays, HEBON, Frans B. L. Hogervorst, Peter J. Hulick, Evgeny N. Imyanitov, Claudine Isaacs, Louise Izatt, Anna Jakubowska, Paul James, Ramunas Janavicius, Uffe Birk Jensen, Esther M. John, Vijai Joseph, Walter Just, Katarzyna Kaczmarek, Beth Y. Karlan, KConFab Investigators, Carolien M. Kets, Judy Kirk, Mieke Kriege, Yael Laitman, Maïté Laurent, Conxi Lazaro, Goska Leslie, Jenny Lester, Fabienne Lesueur, Annelie Liljegren, Niklas Loman, Jennifer T. Loud, Siranoush Manoukian, Milena Mariani, Sylvie Mazoyer, Lesley McGuffog, Hanne E. J. Meijers-Heijboer, Alfons Meindl, Austin Miller, Marco Montagna, Anna Marie Mulligan, Katherine L. Nathanson, Susan L. Neuhausen, Heli Nevanlinna, Robert L. Nussbaum, Edith Olah, Olufunmilayo I. Olopade, Kai-ren Ong, Jan C. Oosterwijk, Ana Osorio, Laura Papi, Sue Kyung Park, Inge Sokilde Pedersen, Bernard Peissel, Pedro Perez Segura, Paolo Peterlongo, Catherine M. Phelan, Paolo Radice, Johanna Rantala, Christine Rappaport-Fuerhauser, Gad Rennert, Andrea Richardson, Mark Robson, Gustavo C. Rodriguez, Matti A. Rookus, Rita Katharina Schmutzler, Nicolas Sevenet, Payal D. Shah, Christian F. Singer, Thomas P. Slavin, Katie Snape, Johanna Sokolowska, Ida Marie Heeholm Sønderstrup, Melissa Southey, Amanda B. Spurdle, Zsofia Stadler, Dominique Stoppa-Lyonnet, Grzegorz Sukiennicki, Christian Sutter, Yen Tan, Muy-Kheng Tea, Manuel R. Teixeira, Alex Teulé, Soo-Hwang Teo, Mary Beth Terry, Mads Thomassen, Laima Tihomirova, Marc Tischkowitz, Silvia Tognazzo, Amanda Ewart Toland, Nadine Tung, Ans M. W. van den Ouweland, Rob B. van der Luijt, Klaartje van Engelen, Elizabeth J. van Rensburg, Raymonda Varon-Mateeva, Barbara Wappenschmidt, Juul T. Wijnen, Timothy Rebbeck, Georgia Chenevix-Trench, Kenneth Offit, Fergus J. Couch, Silje Nord, Douglas F. Easton, Antonis C. Antoniou, Jacques Simard

**Affiliations:** 1 Genomics Center, Centre Hospitalier Universitaire de Québec Research Center and Laval University, 2705 Laurier Boulevard, Quebec, QC G1V 4G2 Canada; 2 Centre for Cancer Genetic Epidemiology, Department of Public Health and Primary Care, University of Cambridge, Strangeways Research Laboratory, Worts Causeway, Cambridge, UK; 3 The Wellcome Trust Sanger Institute, Wellcome Trust Genome Campus Hinxton, Cambridge, CB10 1HH UK; 4 Department of Human Genetics, McGill University, Montreal, QC H3A 1B1 Canada; 5 McGill University and Genome Quebec Innovation Centre, Montreal, QC H3A 0G1 Canada; 6 Yorkshire Regional Genetics Service, Chapel Allerton Hospital, Leeds, LS7 4SA UK; 7 Department of Clinical Genetics, Helsinki University Hospital, HUS, Meilahdentie 2, P.O. BOX 160, 00029 Helsinki, Finland; 8 Lunenfeld-Tanenbaum Research Institute, Mount Sinai Hospital, Toronto, ON M5G 1X5 Canada; 9 Departments of Molecular Genetics and Laboratory Medicine and Pathobiology, University of Toronto, Toronto, ON Canada; 10 Department of Pathology hus 9, Landspitali-LSH v/Hringbraut, 101 Reykjavík, Iceland; 11 BMC (Biomedical Centre), Faculty of Medicine, University of Iceland, Vatnsmyrarvegi 16, 101 Reykjavík, Iceland; 12 Department of Gynaecology and Obstetrics, University Hospital of Schleswig-Holstein, Christian-Albrechts University Kiel, Campus Kiel, 24105 Kiel, Germany; 13 Department of Breast Medical Oncology and Clinical Cancer Genetics Program, University of Texas MD Anderson Cancer Center, 1515 Pressler Street CBP 5, Houston, TX 77030 USA; 14 Unit of Medical Genetics, Department of Preventive and Predictive Medicine, Fondazione IRCCS (Istituto Di Ricovero e Cura a Carattere Scientifico) Istituto Nazionale Tumori (INT), Via Giacomo Venezian 1, 20133 Milan, Italy; 15 Department of Pathology & Molecular Medicine, Juravinski Hospital and Cancer Centre, McMaster University, 711 Concession Street, Hamilton, ON L8V 1C3 Canada; 16 Bâtiment Cheney D, Centre Léon Bérard, 28 rue Laënnec, 69373 Lyon, France; 17 Human Genetics Group, Spanish National Cancer Centre (CNIO), Madrid, Spain; 18 Biomedical Network on Rare Diseases (CIBERER), 28029 Madrid, Spain; 19 Human Genotyping (CEGEN) Unit, Human Cancer Genetics Program, Spanish National Cancer Research Centre (CNIO), Madrid, Spain; 20 Centre François Baclesse, 3 avenue Général Harris, 14076 Caen, France; 21 Department of Clinical Genetics, Maastricht University Medical Center, P.O. Box 5800, 6202 AZ Maastricht, The Netherlands; 22 City of Hope Clinical Cancer Genomics Community Research Network, 1500 East Duarte Road, Duarte, CA 91010 USA; 23 Unité de Prévention et d’Epidémiologie Génétique, Centre Léon Bérard, 28 rue Laënnec, 69373 Lyon, France; 24 Division of Cancer Prevention and Genetics, Istituto Europeo di Oncologia (IEO), Via Ripamonti 435, 20141 Milan, Italy; 25 Department of Medicine, Abramson Cancer Center, Perelman School of Medicine at the University of Pennsylvania, 3400 Civic Center Boulevard, Philadelphia, PA 19104 USA; 26 Department of Clinical Genetics, Royal Devon & Exeter Hospital, Exeter, EX1 2ED UK; 27 Service de Génétique Oncologique, Institut Curie, 26 rue d’Ulm, 75248 Paris Cedex 05, France; 28 Department of Medicine, Huntsman Cancer Institute, 2000 Circle of Hope, Salt Lake City, UT 84112 USA; 29 Section of Genetic Oncology, Department of Laboratory Medicine, University and University Hospital of Pisa, Pisa, Italy; 30 Unité de recherche en santé des populations, Centre des maladies du sein Deschênes-Fabia, Hôpital du Saint-Sacrement, 1050 chemin Sainte-Foy, Quebec, QC G1S 4L8 Canada; 31 Departments of Pediatrics and Medicine, Columbia University, 1150 St. Nicholas Avenue, New York, NY 10032 USA; 32 Center for Medical Genetics, Ghent University, De Pintelaan 185, 9000 Ghent, Belgium; 33 Division of Population Science, Fox Chase Cancer Center, 333 Cottman Avenue, Philadelphia, PA 19111 USA; 34 Department of Clinical Genetics, South Glasgow University Hospitals, Glasgow, G51 4TF UK; 35 Molecular Oncology Laboratory, Hospital Clinico San Carlos, IdISSC (El Instituto de Investigación Sanitaria del Hospital Clínico San Carlos), Martin Lagos s/n, Madrid, Spain; 36 Oncogenetics Group, Vall d’Hebron Institute of Oncology (VHIO), Vall d’Hebron University Hospital, Clinical and Molecular Genetics Area, Passeig Vall d’Hebron 119-129, 08035 Barcelona, Spain; 37 Department of Population Sciences, Beckman Research Institute of City of Hope, Duarte, CA USA; 38 Cancer Bioimmunotherapy Unit, Department of Medical Oncology, Centro di Riferimento Oncologico, IRCCS (Istituto Di Ricovero e Cura a Carattere Scientifico) National Cancer Institute, Via Franco Gallini 2, 33081 Aviano, PN Italy; 39 University of Queensland Diamantina Institute, Translational Research Institute, Brisbane, QLD Australia; 40 Cancer Genetics Laboratory, Department of Genetics, University of Pretoria, Private Bag X323, Arcadia, 0007 South Africa; 41 Faculty of Medicine, University of Southampton, Southampton University Hospitals NHS Trust, Southampton, UK; 42 Oncogenetics Team, The Institute of Cancer Research and Royal Marsden NHS Foundation Trust, Sutton, SM2 5NG UK; 43 Department of Oncology, Sahlgrenska University Hospital, 41345 Göteborg, Sweden; 44 Department of Oncology, Rigshospitalet, Copenhagen University Hospital, Blegdamsvej 9, 2100 Copenhagen, Denmark; 45 Institute for Medical Informatics, Statistics and Epidemiology, University of Leipzig, 04107 Leipzig, Germany; 46 LIFE, Leipzig Research Centre for Civilization Diseases, University of Leipzig, Leipzig, Germany; 47 Genomic Medicine, Manchester Academic Health Sciences Centre, Institute of Human Development, Manchester University, Central Manchester University Hospitals, NHS Foundation Trust, Manchester, M13 9WL UK; 48 Molecular Diagnostic Unit, Hereditary Cancer Program, IDIBELL (Bellvitge Biomedical Research Institute), Catalan Institute of Oncology, Gran Via de l’Hospitalet, 199-203, L’Hospitalet, 08908 Barcelona, Spain; 49 Department of Cancer Epidemiology and Genetics, Masaryk Memorial Cancer Institute, Zluty kopec 7, 65653 Brno, Czech Republic; 50 Molecular Diagnostics Laboratory, (INRASTES) Institute of Nuclear and Radiological Sciences and Technology, National Centre for Scientific Research “Demokritos”, Patriarchou Gregoriou & Neapoleos str., Aghia Paraskevi Attikis, Athens, Greece; 51 Program in Cancer Genetics, Departments of Human Genetics and Oncology, McGill University, Montreal, QC Canada; 52 Department of Medical Oncology, Papageorgiou Hospital, Aristotle University of Thessaloniki School of Medicine, Thessaloníki, Greece; 53 The Susanne Levy Gertner Oncogenetics Unit, Institute of Human Genetics, Chaim Sheba Medical Center, 52621 Ramat Gan, Israel; 54 Sackler Faculty of Medicine, Tel Aviv University, 69978 Ramat Aviv, Israel; 55 Clinical Cancer Genetics, City of Hope, 1500 East Duarte Road, Duarte, CA 91010 USA; 56 UCLA Schools of Medicine and Public Health, Division of Cancer Prevention & Control Research, Jonsson Comprehensive Cancer Center, 650 Charles Young Drive South, Room A2-125 HS, Los Angeles, CA 90095-6900 USA; 57 Cancer Risk and Prevention Clinic, Dana-Farber Cancer Institute, 450 Brookline Avenue, Boston, MA USA; 58 Department of Preventive Medicine, Keck School of Medicine, University of Southern California, Los Angeles, CA 90033 USA; 59 Department of Tumour Biology, Institut Curie, Paris, France; 60 Institut Curie, INSERM U830, Paris, France; 61 Université Paris Descartes, Sorbonne Paris Cité, Paris, France; 62 Department of Clincial Genetics, Rigshospitalet, Blegdamsvej 9, 4062 Copenhagen, Denmark; 63 Department of Pathology and Laboratory Medicine, University of Kansas Medical Center, 3901 Rainbow Boulevard, 4019 Wahl Hall East, MS 3040, Kansas City, Kansas USA; 64 Department of Dermatology, University of Utah School of Medicine, 30 North 1900 East, SOM 4B454, Salt Lake City, UT 84132 USA; 65 Clinical Genetics Branch, DCEG, NCI NIH, 9609 Medical Center Drive, Room 6E-454, Bethesda, MD USA; 66 Department of Genetics and Pathology, Pomeranian Medical University, Polabska 4, 70-115 Szczecin, Poland; 67 Centre of Familial Breast and Ovarian Cancer, Department of Gynaecology and Obstetrics and Centre for Integrated Oncology (CIO), Center for Molecular Medicine Cologne (CMMC), University Hospital of Cologne, 50931 Cologne, Germany; 68 Molecular Genetics of Breast Cancer, German Cancer Research Center (DKFZ), Im Neuenheimer Feld 580, 69120 Heidelberg, Germany; 69 Center for Genomic Medicine, Rigshospitalet, Copenhagen University Hospital, Blegdamsvej 9, 2100 Copenhagen, Denmark; 70 Department of Health Sciences Research, Mayo Clinic, 200 First Street SW, Rochester, MN 55905 USA; 71 Division of Medical Oncology, Department of Internal Medicine, The Ohio State University, Columbus, OH 43210 USA; 72 Division of Gynecologic Oncology, Department of Obstetrics and Gynecology, The Ohio State University, Columbus, OH 43210 USA; 73 Comprehensive Cancer Center Arthur C. James Cancer Hospital and Richard J. Solove Research Institute Biomedical Research Tower, Room 588, 460 West 12th Avenue, Columbus, OH 43210 USA; 74 The Hereditary Breast and Ovarian Cancer Research Group Netherlands (HEBON), Coordinating Center: Netherlands Cancer Institute, Amsterdam, The Netherlands; 75 Family Cancer Clinic, Netherlands Cancer Institute, P.O. Box 90203, 1006 BE Amsterdam, The Netherlands; 76 Center for Medical Genetics, NorthShore University HealthSystem, University of Chicago Pritzker School of Medicine, 1000 Central Street, Suite 620, Evanston, IL 60201 USA; 77 N.N. Petrov Institute of Oncology, St. Petersburg, Russia 197758; 78 Lombardi Comprehensive Cancer Center, Georgetown University, 3800 Reservoir Road NW, Washington, DC 20007 USA; 79 Clinical Genetics, Guy’s and St. Thomas’ NHS Foundation Trust, London, SE1 9RT UK; 80 Familial Cancer Centre, Peter MacCallum Cancer Centre, Melbourne, VIC 3000 Australia; 81 Sir Peter MacCallum Department of Oncology, University of Melbourne, Melbourne, VIC 3010 Australia; 82 Hematology, Oncology and Transfusion Medicine Center, Department of Molecular and Regenerative Medicine, Vilnius University Hospital Santariskiu Clinics, Santariskiu st. 2, 08661 Vilnius, Lithuania; 83 State Research Institute Centre for Innovative Medicine, Zygymantu st. 9, Vilnius, Lithuania; 84 Department of Clinical Genetics, Aarhus University Hospital, Brendstrupgaardsvej 21C, Århus N, Denmark; 85 Department of Epidemiology, Cancer Prevention Institute of California, 2201 Walnut Avenue Suite 300, Fremont, CA 94538 USA; 86 Department of Health Research and Policy (Epidemiology) and Stanford Cancer Institute, Stanford University School of Medicine, Stanford, CA USA; 87 Clinical Genetics Research Laboratory, Department of Medicine, Memorial Sloan-Kettering Cancer Center, 1275 York Avenue, New York, NY 10044 USA; 88 Institute of Human Genetics, University of Ulm, 89091 Ulm, Germany; 89 Women’s Cancer Program at the Samuel Oschin Comprehensive Cancer Institute, Cedars-Sinai Medical Center, 8700 Beverly Boulevard, Suite 290W, Los Angeles, CA 90048 USA; 90 Research Department, Peter MacCallum Cancer Centre, East Melbourne, Melbourne, VIC 8006 Australia; 91 Department of Human Genetics, Radboud University Medical Center, P.O. Box 9101, 6500 HB Nijmegen, The Netherlands; 92 Westmead Hospital, Familial Cancer Service, Hawkebury Road, P.O. Box 533, Wentworthville, NSW 2145 Australia; 93 Department of Medical Oncology, Family Cancer Clinic, Erasmus University Medical Center, P.O. Box 5201, 3008 AE Rotterdam, The Netherlands; 94 Genetic Epidemiology of Cancer Team, INSERM U900, Institut Curie Mines ParisTech, PSL University, 26 rue d’Ulm, 75248 Paris Cedex 05, France; 95 Department of Oncology, Karolinska University Hospital, 17176 Stockholm, Sweden; 96 Department of Oncology, Lund University Hospital, 22185 Lund, Sweden; 97 Lyon Neuroscience Research Center-CRNL, INSERM U1028, CNRS UMR5292, University of Lyon, Lyon, France; 98 Department of Clinical Genetics, VU University Medical Center, P.O. Box 7057, 1007 MB Amsterdam, The Netherlands; 99 NRG Oncology Statistics and Data Management Center, Roswell Park Cancer Institute, Elm St & Carlton St, Buffalo, NY 14263 USA; 100 Immunology and Molecular Oncology Unit, Veneto Institute of Oncology IOV-IRCCS, Via Gattamelata 64, 35128 Padua, Italy; 101 Department of Laboratory Medicine and the Keenan Research Centre of the Li Ka Shing Knowledge Institute, St Michael’s Hospital, Toronto, ON Canada; 102 Department of Obstetrics and Gynecology, University of Helsinki and Helsinki University Hospital, Biomedicum Helsinki, Haartmaninkatu 8, HUS, P.O. BOX 700, 00029 Helsinki, Finland; 103 Department of Medicine and Genetics, University of California, 513 Parnassus Ave., HSE 901E, San Francisco, CA 94143-0794 USA; 104 Department of Molecular Genetics, National Institute of Oncology, Budapest, Hungary; 105 Department of Medicine, University of Chicago, 5841 South Maryland Avenue, MC 2115, Chicago, IL USA; 106 West Midlands Regional Genetics Service, Birmingham Women’s Hospital Healthcare NHS Trust, Edgbaston, Birmingham, UK; 107 Department of Genetics, University Medical Center Groningen, University of Groningen, 9700 RB Groningen, The Netherlands; 108 Unit of Medical Genetics, Department of Biomedical Experimental and Clinical Sciences, University of Florence, Viale Morgagni 50, 50134 Florence, Italy; 109 Department of Preventive Medicine, Seoul National University College of Medicine, 103 Daehak-ro, Jongno-gu, Seoul, 110-799 Korea; 110 Section of Molecular Diagnostics, Department of Biochemistry, Aalborg University Hospital, Reberbansgade 15, Ålborg, Denmark; 111 Department of Oncology, Hospital Clinico San Carlos, IdISSC (El Instituto de Investigación Sanitaria del Hospital Clínico San Carlos), Martin Lagos s/n, Madrid, Spain; 112 IFOM, The FIRC (Italian Foundation for Cancer Research) Institute of Molecular Oncology, c/o IFOM-IEO Campus, Via Adamello 16, 20139 Milan, Italy; 113 Department of Cancer Epidemiology, Moffitt Cancer Center, Tampa, FL 33612 USA; 114 Unit of Molecular Bases of Genetic Risk and Genetic Testing, Department of Preventive and Predicted Medicine, Fondazione IRCCS (Istituto Di Ricovero e Cura a Carattere Scientifico) Istituto Nazionale Tumori (INT), c/o Amaedeolab via GA Amadeo 42, 20133 Milan, Italy; 115 Department of Clinical Genetics, Karolinska University Hospital, L5:03, 171 76 Stockholm, Sweden; 116 Department of OB/GYN, Medical University of Vienna, Waehringer Guertel 18-20, A, 1090 Vienna, Austria; 117 Clalit National Israeli Cancer Control Center and Department of Community Medicine and Epidemiology, Carmel Medical Center and B. Rappaport Faculty of Medicine, 7 Michal St., 34362 Haifa, Israel; 118 Department of Pathology, Johns Hopkins University School of Medicine, Baltimore, MD 21205 USA; 119 Clinical Genetics, Services Department of Medicine, Memorial Sloan-Kettering Cancer Center, 1275 York Avenue, New York, NY 10065 USA; 120 Division of Gynecologic Oncology, NorthShore University HealthSystem, University of Chicago, 2650 Ridge Avenue, Suite 1507, Walgreens, Evanston, IL 60201 USA; 121 Department of Epidemiology, Netherlands Cancer Institute, P.O. Box 90203, 1006 BE Amsterdam, The Netherlands; 122 Center for Hereditary Breast and Ovarian Cancer, Medical Faculty, University Hospital Cologne, 50931 Cologne, Germany; 123 Center for Integrated Oncology (CIO), Medical Faculty, University Hospital Cologne, Cologne, Germany; 124 Oncogénétique, Institut Bergonié, 229 cours de l’Argonne, 33076 Bordeaux, France; 125 Medical Genetics Unit, St George’s, University of London, London, SW17 0RE UK; 126 Laboratoire de génétique médicale Nancy Université, Centre Hospitalier Régional et Universitaire, Rue du Morvan cedex 1, 54511 Vandoeuvre-les-Nancy, France; 127 Department of Pathology Region Zealand Section Slagelse, Slagelse Hospital, Ingemannsvej 18 Slagelse, Cpoenhagen, Denmark; 128 Genetic Epidemiology Laboratory, Department of Pathology, University of Melbourne, Parkville, VIC 3010 Australia; 129 Genetics and Computational Biology Department, QIMR Berghofer Medical Research Institute, Herston Road, Brisbane, QLD 4006 Australia; 130 Clinical Genetics Service, Department of Medicine, Memorial Sloan Kettering Cancer Center, 1275 York Avenue, New York, NY USA; 131 Institute of Human Genetics, Department of Human Genetics, University Hospital Heidelberg, Heidelberg, Germany; 132 Department of Genetics, Portuguese Oncology Institute, Rua Dr. António Bernardino de Almeida, 4200-072 Porto, Portugal; 133 Biomedical Sciences Institute (ICBAS), University of Porto, Porto, Portugal; 134 Genetic Counseling Unit, Hereditary Cancer Program, IDIBELL (Bellvitge Biomedical Research Institute), Catalan Institute of Oncology, Gran Via de l’Hospitalet, 199-203, L’Hospitalet, 08908 Barcelona, Spain; 135 Cancer Research Initiatives Foundation, Sime Darby Medical Centre, 1 Jalan SS12/1A, 47500 Subang Jaya, Malaysia; 136 University Malaya Cancer Research Institute, University Malaya, 1 Jalan SS12/1A, 50603 Kuala Lumpur, Malaysia; 137 Department of Epidemiology, Columbia University, New York, NY USA; 138 Department of Clinical Genetics, Odense University Hospital, Sonder Boulevard 29, Odense C, Denmark; 139 Latvian Biomedical Research and Study Centre, Ratsupites str 1, Riga, Latvia; 140 Department of Medical Genetics Level 6 Addenbrooke’s Treatment Centre, Addenbrooke’s Hospital, Hills Road, Box 134, Cambridge, CB2 0QQ UK; 141 Division of Human Genetics, Departments of Internal Medicine and Cancer Biology and Genetics Comprehensive Cancer Center, The Ohio State University, 998 Biomedical Research Tower, Columbus, OH 43210 USA; 142 Department of Medical Oncology, Beth Israel Deaconess Medical Center, 330 Brookline Avenue, Boston, MA 02215 USA; 143 Department of Clinical Genetics, Family Cancer Clinic, Erasmus University Medical Center, 330 Brookline Avenue, P.O. Box 2040, 3000 CA Rotterdam, The Netherlands; 144 Department of Medical Genetics, University Medical Center Utrecht, 3584 EA Utrecht, The Netherlands; 145 Department of Clinical Genetics, Academic Medical Center, P.O. Box 22700, 1100 DE Amsterdam, The Netherlands; 146 Institute of Human Genetics, Charite Berlin, Campus Virchov Klinikum, 13353 Berlin, Germany; 147 Department of Human Genetics & Department of Clinical Genetics, Leiden University Medical Center, 2300 RC Leiden, The Netherlands; 148 Center for Clinical Epidemiology and Biostatistics, Perelman School of Medicine at the University of Pennsylvania, Philadelphia, PA USA; 149 Department of Laboratory Medicine and Pathology, Mayo Clinic, 200 First Street SW, Rochester, MN 55905 USA; 150 Department of Cancer Genetics, Institute for Cancer Research, Oslo University Hospital, Radiumhospitalet, 0372 Oslo, Norway

**Keywords:** Breast cancer, Genetic modifiers, Differential allelic expression, Genetic susceptibility, *Cis*-regulatory variants, *BRCA1* and *BRCA2* mutation carriers

## Abstract

**Purpose:**

*Cis*-acting regulatory SNPs resulting in differential allelic expression (DAE) may, in part, explain the underlying phenotypic variation associated with many complex diseases. To investigate whether common variants associated with DAE were involved in breast cancer susceptibility among *BRCA1* and *BRCA2* mutation carriers, a list of 175 genes was developed based of their involvement in cancer-related pathways.

**Methods:**

Using data from a genome-wide map of SNPs associated with allelic expression, we assessed the association of ~320 SNPs located in the vicinity of these genes with breast and ovarian cancer risks in 15,252 *BRCA1* and 8211 *BRCA2* mutation carriers ascertained from 54 studies participating in the Consortium of Investigators of Modifiers of *BRCA1/2*.

**Results:**

We identified a region on 11q22.3 that is significantly associated with breast cancer risk in *BRCA1* mutation carriers (most significant SNP rs228595 *p* = 7 × 10^−6^). This association was absent in *BRCA2* carriers (*p* = 0.57). The 11q22.3 region notably encompasses genes such as *ACAT1*, *NPAT*, and *ATM*. Expression quantitative trait loci associations were observed in both normal breast and tumors across this region, namely for *ACAT1*, *ATM*, and other genes. In silico analysis revealed some overlap between top risk-associated SNPs and relevant biological features in mammary cell data, which suggests potential functional significance.

**Conclusion:**

We identified 11q22.3 as a new modifier locus in *BRCA1* carriers. Replication in larger studies using estrogen receptor (ER)-negative or triple-negative (i.e., ER-, progesterone receptor-, and HER2-negative) cases could therefore be helpful to confirm the association of this locus with breast cancer risk.

**Electronic supplementary material:**

The online version of this article (doi:10.1007/s10549-016-4018-2) contains supplementary material, which is available to authorized users.

## Introduction

Pathogenic mutations in the *BRCA1* and *BRCA2* genes substantially increase a woman’s lifetime risk of developing breast and ovarian cancers [1–4]. These risks vary significantly according to (a) age at disease diagnosis in carriers of identical mutations, (b) the cancer site in the individual who led to the family’s ascertainment, (c) the degree of family history of the disease [1, 4, 5], and (d) the type and location of *BRCA1* and *BRCA2* mutations [6]. These observations suggest that other factors, including lifestyle/hormonal factors [7] as well as other genetic factors, modify cancer risks in *BRCA1* and *BRCA2* mutation carriers. Direct evidence for such genetic modifiers of risk has been obtained through the association studies performed by the Consortium of Investigators of Modifiers of *BRCA1*/*2* (CIMBA), which have shown that several common breast cancer susceptibility alleles identified through population-based genome-wide association studies (GWASs) are also associated with breast cancer risk among *BRCA1* and *BRCA2* mutation carriers [8–10].

Global analysis of GWAS data has shown that the vast majority of common variants associated with susceptibility to cancer lie within genomic non-coding regions and are predicted to account for cancer risk through regulation of gene expression [11, 12]. A recent expression quantitative trait loci (*cis*-eQTL) analysis for mRNA expression in 149 known cancer risk loci performed in five tumor types (breast, colon, kidney, lung, and prostate) has shown that approximately 30 % of such risk loci were significantly associated with eQTLs present in at least one gene within 500 kb [13]. These results suggest that additional cancer susceptibility loci may be identified through studying genetic variants that affect the regulation of gene expression. In the present study, we selected genes of interest for their known involvement in cancer etiology, identified 320 genetic variants in the vicinity of these genes with evidence of differential allelic expression (DAE), and then investigated the associations of these variants with breast and ovarian cancer risks among *BRCA1* and *BRCA2* mutation carriers. These included variants in genes involved in DNA repair (homologous recombination and DNA interstrand crosslink repair), interaction with and/or modulation of BRCA1 and BRCA2 cellular functions, cell cycle control, centrosome amplification and interaction with AURKA, apoptosis, ubiquitination, as well as known tumor suppressors, mitotic kinases, and other kinases, sex steroid action, and mammographic density.

## Materials and methods

### Subjects

All study participants were female carriers of a deleterious germline mutation in either *BRCA1* or *BRCA2* and aged 18 years or older [14]. Fifty-four collaborating CIMBA studies contributed a total of 23,463 samples (15,252 *BRCA1* mutation carriers and 8211 *BRCA2* mutation carriers) to this study, including 12,127 with breast cancer (7797 *BRCA1* and 4330 *BRCA2* carriers) and 3093 with ovarian cancer (2462 *BRCA1* and 631 *BRCA2* carriers). The number of samples included from each study is provided in Online Resource 1. The recruitment strategies, clinical, demographic, and phenotypic data collected from each participant have been previously reported [14].

### Ethics statement

*BRCA1* and *BRCA2* mutation carriers were recruited through the CIMBA initiative, following approval of the corresponding protocol by the Institutional Review Board or Ethics Committee at each participating center (Online Resource 2); written informed consent was obtained from all study participants [8, 9].

### SNP selection and differential allelic expression

SNP selection was performed by first identifying a list of 175 genes of interest involved in cancer-related pathways and/or mechanisms. The list of genes was established by analyzing published results and by using available public databases such as the Kyoto encyclopedia of genes and genomes (http://www.genome.jp/kegg/). Next, DAE SNPs located within these gene regions were identified using previously reported data on allelic expression *cis*-associations, derived using (1) the lllumina Human1M-duo BeadChip for lymphoblastoid cell lines from Caucasians (CEU population) (*n* = 53) [15], the Illumina Human 1M Omni-quad for primary skin fibroblasts derived from Caucasian donors (*n* = 62) [13, 16], and the Illumina Infinium II assay with Human 1.2M Duo custom BeadChip v1 for human primary monocytes (*n* = 188) [17]. Briefly, 1000 Genomes project data were used as a reference set (release 1000G Phase I v3) for the imputation of genotypes from HapMap individuals. Genotypes were inferred using algorithms implemented in IMPUTE2 [18]. The unrelated fibroblast panel consisted of 31 parent–offspring trios, in which the genotypes of offspring were used to permit accurate phasing. Mapping of each allelic expression trait was carried out by first normalizing allelic expression ratios at each SNP using a polynomial method [19] and then calculating average phased allelic expression scores across annotated transcripts, followed by correlation of these scores to local (transcript ± 500 kb) SNP genotypes in fibroblasts as described earlier [16]. A total of 355 genetic variants were selected on the basis of evidence of association with DAE in the selected 175 genes (see Online Resource 3 for a complete list of SNPs and genes). Following the selection process, SNPs were submitted for design and inclusion on a custom-made Illumina Infinium array (iCOGS) as previously described [8, 9]. Following probe design and post-genotyping quality control, 316 and 317 SNPs were available for association analysis in *BRCA1* and *BRCA2* mutation carriers, respectively. Genotyping and quality control procedures have been described in detail elsewhere [8, 9].

### Statistical analysis

Associations between genotypes and breast and ovarian cancer risks were evaluated within a survival analysis framework, using a one degree-of-freedom score test statistic based on modeling the retrospective likelihood of the observed genotypes conditional on the disease phenotypes [20, 21]. To estimate the magnitude of the associations [hazard ratios (HRs)], we maximized the retrospective likelihood, which was parameterized in terms of the per-allele HR. All analyses were stratified by country of residence and using calendar year and cohort-specific incidence rates of breast and ovarian cancers for mutation carriers. Given 320 tests, the cutoff value for significance after a Bonferroni adjustment for multiple testing was *p* < 1.5 × 10^−4^.

The associations between the genotypes and tumor subtypes were evaluated using an extension of the retrospective likelihood approach that models the association with two or more subtypes simultaneously [22].

Imputation was performed separately for *BRCA1* and *BRCA2* mutation carriers to estimate genotypes for other common variants across a ±50-kb region centered around the 12 most strongly associated SNPs (following the NCBI Build 37 assembly), using the March 2012 release of the 1000 Genomes Project as the reference panel and the IMPUTE v.2.2 software [18]. In all analyses, only SNPs with an imputation accuracy coefficient *r* ^2^ >0.30 were considered [8, 9].

### Functional annotation

Publicly available genomic data were used to annotate the SNPs most strongly associated with breast cancer risk at locus 11q22.3. The following regulatory features were obtained for breast cell types from ENCODE and NIH Roadmap Epigenomics data through the UCSC Genome Browser: DNase I hypersensitivity sites, chromatin hidden Markov modeling (ChromHMM) states, and histone modifications of epigenetic markers, more specifically commonly used marks associated with enhancers (H3K4Me1 and H3K27Ac) and promoters (H3K4Me3 and H3K9Ac). To identify putative target genes, we examined potential functional chromatin interactions between distal and proximal regulatory transcription factor-binding sites and the promoters at the risk loci, using the chromatin interaction analysis by paired end tag (ChiA-PET) and genome conformation capture (Hi-C, 3C, and 5C) datasets downloaded from GEO and from 4D-genome [23]. Maps of active mammary super-enhancer regions in human mammary epithelial cells (HMECs) were obtained from Hnisz et al. [24]. Enhancer–promoter specific interactions were predicted from the integrated method for predicting enhancer targets (IM-PETs) [25]. RNA-Seq data from ENCODE was used to evaluate the expression of exons across the 11q22.3 locus in MCF7 and HMEC cell lines. For MCF7 and HMEC, alignment files from 19 and 4 expression datasets, respectively, were downloaded from ENCODE using a rest API wrapper (ENCODExplorer R package) [26] in the bam format and processed using metagene R packages [27] to normalize in Reads per Millions aligned and to convert into coverages.

### eQTL analyses

The influence of germline genetic variations on gene expression was assessed using a linear regression model, as implemented in the R library eMAP (http://www.bios.unc.edu/~weisun/software.htm). An additive effect was inferred by modeling subjects’ copy number of the rare allele, i.e., 0, 1, or 2 for a given genotype. Only relationships in *cis* (defined as those for which the SNP is located at <1 Mb upstream or downstream from the center of the transcript) were investigated. The eQTL analyses were performed on both normal and tumor breast tissues (see Online Resource 4 for the list and description of datasets, as well as the sources of genotype and expression data). For all sample sets, the genotyping data were processed as follows: SNPs with call rates <0.95 or minor allele frequencies, MAFs (<0.05) were excluded, as were SNPs out of Hardy–Weinberg equilibrium with *P* < 10^−13^. All samples with a call rate <80 % were excluded. Identity by state was computed using the R GenABEL package [28], and samples from closely related individuals whose identity by state was lower than 0.95 were removed. The SNP and sample filtration criteria were applied iteratively until all samples and SNPs met the set thresholds.

## Results

From the 175 genes selected for their involvement in cancer-related pathways and/or mechanisms, we identified a set of 355 genetic variants showing evidence of association with DAE (see Online Resource 3 for the complete list of genes and SNPs). Of those, 39 and 38 SNPs were excluded because of low Illumina design scores, low call rates, and/or evidence of deviation from Hardy–Weinberg equilibrium (*P* value <10^−7^), for *BRCA1* and *BRCA2* analyses, respectively. A total of 316 and 317 SNPs (representing 227 independent SNPs with a pairwise *r* ^2^ <0.1) were successfully genotyped in 15,252 *BRCA1* and 8211 *BRCA2* mutation carriers, respectively. Association results for breast and ovarian cancer risks for all SNPs are presented in Online Resource 5.

### Breast cancer association analysis

Evidence of association with breast cancer risk (at *p* < 10^−2^) was observed for nine SNPs in *BRCA1* mutation carriers and three SNPs in *BRCA2* mutation carriers (Table 1). The strongest association with breast cancer risk among *BRCA1* carriers was observed for rs6589007, located at 11q22.3 in intron 15 of the *NPAT* gene (*p* = 4.6 × 10^−3^) at approximately 54 kb upstream of the *ATM* gene. Similar associations were observed for two other highly correlated variants (*r* ^2^ >0.8) on chromosome 11, namely rs183459 (*p* = 5.7 × 10^−3^) also located within *NPAT* and rs228592 (*p* = 5.5 × 10^−3^) located in intron 11 of *ATM*. No association was observed between SNPs at this locus and breast cancer risk for *BRCA2* carriers (Online Resource 5).

**Table 1:** Associations with breast cancer risk in *BRCA1* and *BRCA2* mutation carriers for SNPs observed at *p* < 10^−2^

Locations	Positions	SNPs	Nearest genes	Unaffected (number)	Affected (number)	Unaffected (MAF)	Affected (MAF)	HR* (95 % CI)	*p* values
*BRCA1* mutation carriers									
1q42.13	227,308,416	rs11806633	*CDC42BPA*	7455	7797	0.07	0.06	1.128 (1.039–1.225)	4.8 × 10^−3^
2p23.2	28,319,320	rs6721310	*BRE*	7454	7793	0.33	0.33	1.064 (1.018–1.111)	5.4 × 10^−3^
2q11.2	100,019,496	rs2305354	*REV1*	7451	7796	0.44	0.45	1.057 (1.015–1.100)	7.1 × 10^−3^
4p15.33	14,858,341	rs1389999	*CEBP*	7454	7795	0.35	0.35	0.940 (0.901–0.982)	5.3 × 10^−3^
5q14.1	79,901,952	rs425463	*DHFR*, *MSH3*	7430	7755	0.33	0.35	1.058 (1.013–1.105)	9.5 × 10^−3^
11q22.3	108,040,104	rs6589007	*NPAT*, *ACAT1*, *ATM*	7451	7797	0.41	0.42	1.062 (1.019–1.107)	4.6 × 10^−3^
11q22.3	108,089,197	rs183459	*NPAT*, *ATM*	7447	7789	0.40	0.41	1.061 (1.018–1.105)	5.7 × 10^−3^
11q22.3	108,123,189	rs228592	*ATM*	7449	7792	0.42	0.41	1.061 (1.018–1.106)	5.5 × 10^−3^
12p13.33	986,004	rs7967755	*WNK1*, *RAD52*	7454	7797	0.16	0.152	0.927 (0.876–0.980)	7.5 × 10^−3^
*BRCA2* mutation carriers									
6p22.1	28,231,243	rs9468322	*NKAPL*	3880	4329	0.04	0.05	1.235 (1.080–1.412)	4.2 × 10^−3^
8q11.21	48,708,742	rs6982040	*PRKDC*	3876	4327	0.006	0.002	0.497 (0.292–0.843)	2.7 × 10^−3^
16p13.3	1,371,154	rs2268049	*UBE2I*	3880	4325	0.14	0.16	1.116 (1.031–1.207)	4.5 × 10^−3^

*CI* confidence interval, *HR* hazard ratio, *MAF* minor allele frequency, *SNP* single-nucleotide polymorphism

* Hazard ratio per allele (one degree of freedom) estimated from the retrospective likelihood analysis

The strongest evidence of association with breast cancer risk in *BRCA2* mutation carriers was observed for rs6982040, located at 8q11.21 in intron 74 of the *PRKDC* gene (*p* = 2.7 × 10^−3^). However, this variant had a very low frequency in affected and unaffected individuals (MAF values of 0.002 and 0.006, respectively). No association was observed for this locus in *BRCA1* carriers (Online Resource 5).

Of the nine SNPs associated with breast cancer risk in *BRCA1* mutation carriers, three were primarily associated with estrogen receptor (ER)-negative breast cancer: rs11806633 at 1q42.13 in the *CDC42BPA* gene (*p* = 9.0 × 10^−3^), rs6721310 at 2p23.2 in the *BRE* gene (*p* = 3.0 × 10^−3^), and rs2305354 at 2q11.2 in the *REV1* gene (*p* = 1.0 × 10^−3^), although the differences between ER-positive and ER-negative disease associations were not statistically significant (Table 2). Of the three *BRCA2*-associated loci, only rs9468322 at 6p22.1 was associated with ER-positive disease (*p* = 5.0 × 10^−4^), although the differences in HRs between ER-positive and ER-negative tumors were not statistically significant (Table 2).

**Table 2:** Associations with breast cancer risk by tumor subtype in *BRCA1* and *BRCA2* mutation carriers

Locations	Positions	SNPs	ER-positive		ER-negative		ER-diff
			HR (95 % CI)	*p* values	HR (95 % CI)	*p* values	*p*-diff
*BRCA1* mutation carriers							
1q42.13	227,308,416	rs11806633	1.10 (0.90–1.33)	0.35	1.14 (1.03–1.25)	9.0 × 10^−3^	0.73
2p23.2	28,319,320	rs6721310	1.00 (0.88–1.09)	0.96	1.08 (1.04–1.15)	3.0 × 10^−3^	0.20
2q11.2	100,019,496	rs2305354	0.98 (0.91–1.10)	0.71	1.09 (1.03–1.13)	1.0 × 10^−3^	0.09
4p15.33	14,858,341	rs1389999	0.94 (0.85–1.04)	0.20	0.94 (0.89–0.99)	2.0 × 10^−2^	0.95
5q14.1	79,901,952	rs425463	1.04 (0.94–1.15)	0.48	1.07 (1.01–1.12)	1.6 × 10^−2^	0.67
11q22.3	108,040,104	rs6589007	1.08 (0.99–1.19)	9.8 × 10^−2^	1.06 (1.01–1.11)	2.0 × 10^−2^	0.66
11q22.3	108,089,197	rs183459	1.08 (0.99–1.19)	9.3 × 10^−2^	1.05 (1.00–1.11)	3.7 × 10^−2^	0.62
11q22.3	108,123,189	rs228592	1.08 (0.96–1.19)	9.7 × 10^−2^	1.06 (1.00–1.11)	3.4 × 10^−2^	0.64
12p13.33	986,004	rs7967755	0.96 (0.84–1.09)	0.54	0.92 (0.86–0.98)	1.0 × 10^−2^	0.56
*BRCA2* mutation carriers							
6p22.1	28,231,243	rs9468322	1.30 (1.12–1.51)	5.0 × 10^−4^	1.00 (0.72–1.40)	0.99	0.17
8q11.21	48,708,742	rs6982040	N/A	N/A	N/A	N/A	N/A
16p13.3	1,371,154	rs2268049	1.10 (1.01–1.21)	4.0 × 10^−2^	1.17 (0.98–1.39)	8.0 × 10^−2^	0.56

*CI* confidence interval, *HR* hazard ratio, *SNP* single-nucleotide polymorphism, *N*/*A* not available

* Hazard ratio per allele (one degree of freedom) estimated from the retrospective likelihood analysis

**Fig. 1:**
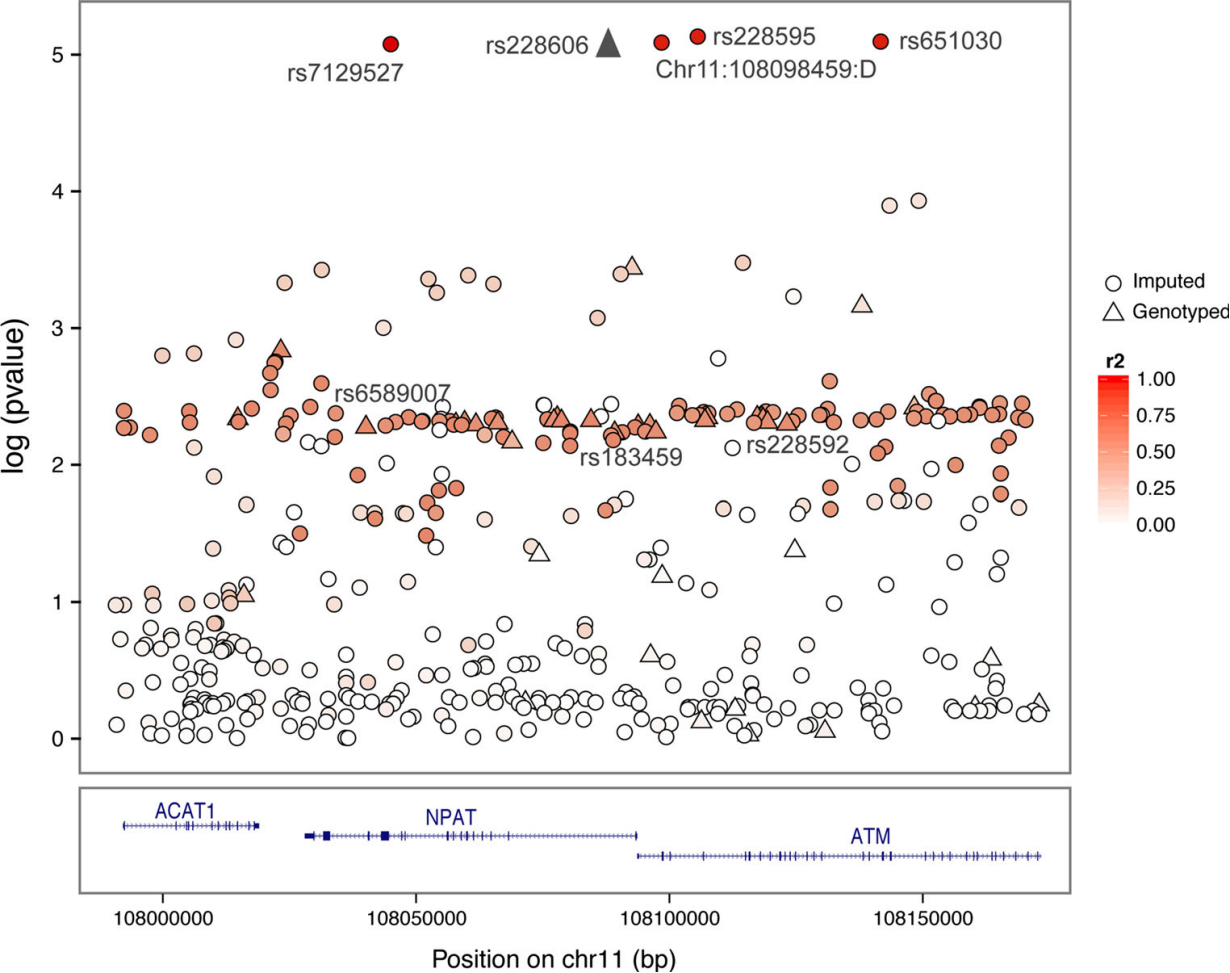
Manhattan plot depicting the strength of association between breast cancer risk in *BRCA1* mutation carriers and all imputed and genotyped SNPs located across the 11q22.3 locus bound by hg19 coordinates chr11:107990104_108173189. Directly genotyped SNPs are represented as *triangles* and imputed SNPs (*r* ^2^ > 0.3, MAF > 0.02) are represented as *circles*. The linkage disequilibrium (*r* ^2^) for the most strongly associated genotyped SNP with each SNP was computed based on subjects of European ancestry that were included in the 1000 Genome Mar 2012 EUR release. Pairwise *r* ^2^ values are plotted using a *red* scale, where *white* and *red* means *r* ^2^ = 0 and 1, respectively. SNPs are plotted according to their chromosomal position: physical locations are based on the GRCh37/hg19 map. SNP rs228606 was genotyped in the iCOGS array but was not included in our original hypothesis of association with DAE. Gene annotation is based on the NCBI RefSeq gene descriptors from the UCSC genome browser

Although evidence of association with breast cancer risk was observed for the above-described loci in *BRCA1* and *BRCA2* mutation carriers, none of these associations reached significance after a Bonferroni adjustment for multiple testing. Imputation using the 1000 Genomes data (encompassing ± 50 kb centered on each of the 12 associated variants, Online Resource 6) identified several SNPs with significant associations in *BRCA1* mutation carriers at the 11q22.3 locus (with SNP rs228595 as the most significant, *p* = 7.38 × 10^−6^), and which were partly correlated with the genotyped SNPs (*r* ^2^ <0.4, Fig. 1). After imputation, we also found associations (albeit not statistically significant after multiple testing adjustments), between one imputed SNP at locus 12p13 (rs2255390, *p* = 5.0 × 10^−4^) and breast cancer risk for *BRCA1* carriers, and two SNPs and breast cancer risk for *BRCA2* carriers, namely 6p22 (chr6:28226644:I, *p* = 9.0 × 10^−4^) and 8q11 (rs189286892, *p* = 2.0 × 10^−4^).

### Ovarian cancer association analyses

Evidence of association with ovarian cancer risk (*p* < 10^−2^) was observed for six SNPs in *BRCA1* mutation carriers and three SNPs in *BRCA2* mutation carriers (Table 3). The strongest association with ovarian cancer risk in *BRCA1* carriers was observed for rs12025623 located at 1p36.12 (*p* = 7 × 10^−3^) in an intron of the *ALPL* gene. Another correlated variant (*r* ^2^ >0.7) on chromosome 1 was also genotyped, namely rs1767429 (*p* = 9 × 10^−3^), which was also located within *ALPL.* The strongest evidence of association with ovarian cancer risk in *BRCA2* mutation carriers was observed for rs2233025 (*p* = 5 × 10^−3^), located at 1p32.22 within the *MAD2L2* gene. None of these associations remained statistically significant after multiple testing adjustments. Imputed genotypes of SNPs in a region encompassing ± 50 kb centered on each of the nine associated variants did not identify stronger associations.

**Table 3:** Associations with ovarian cancer risk in *BRCA1* and *BRCA2* mutation carriers for SNPs observed at *p* < 10^−2^

Locations	Positions	SNPs	Nearest genes	Unaffected (number)	Affected (number)	Unaffected (MAF)	HR* (95 % CI)	*p* values
*BRCA1* mutation carriers								
1p36.12	21,889,340	rs1767429	*ALPL*, *RAP1GAP*	12,765	2460	0.42	1.092 (1.024–1.164)	9 × 10^−3^
1p36.12	21,892,479	rs12025623	*ALPL*, *RAP1GAP*	12,789	2460	0.36	1.098 (1.027–1.173)	7 × 10^−3^
6p21.32	32,913,246	rs1480380	*BRD2*, *HLA*-*DMB*, *HLA*-*DMA*	12,790	2462	0.07	1.178 (1.041–1.333)	9 × 10^−3^
10p12.1	27,434,716	rs788209	*ANKRD26*, *YME1L1*, *MASTL*, *ACBD5*	12,754	2455	0.15	0.879 (0.804–0.961)	5 × 10^−3^
17p13.1	8,071,592	rs3027247	*MIR3676*, *C17orf59*, *AURKB*, *C17orf44*, *C17orf68*, *PFAS*	12,786	2461	0.29	0.905 (0.844–0.970)	5 × 10^−3^
17q22	53,032,425	rs17817865	*MIR4315*-*1*, *TOM1L1*, *COX11*, *STXBP4*	12,790	2462	0.27	0.905 (0.842–0.971)	8 × 10^−3^
*BRCA2* mutation carriers								
1p32.22	11,735,652	rs2233025	*MAD2L2*, *FBXO6*	7574	631	0.18	0.777 (0.657–0.919)	5 × 10^−3^
9p13.3	35,055,669	rs595429	*VCP*, *FANCG*, *c9orf131*	7579	631	0.46	0.856 (0.758–0.964)	6 × 10^−3^
17q25.3	76,219,783	rs2239680	*DHX29*, *SKIV2L2*	7579	630	0.28	0.828 (0.722–0.948)	7 × 10^−3^

*CI* confidence interval, *HR* hazard ratio, *MAF* minor allele frequency, *SNP* single-nucleotide polymorphism

* Hazard ratio per allele (one degree of freedom) estimated from the retrospective likelihood analysis

### eQTL analysis in breast tissue

To identify the genes influenced via the observed associations with breast cancer at locus 11q22.3, eQTL analysis was performed using gene expression data from tumor and normal breast tissues (for detailed descriptions of datasets, refer to Online Resource 4), and all genotyped as well as imputed SNPs within a 1-Mb region on either side of the most significant genotyped SNP. eQTL associations were observed in both normal and tumor breast tissues in this region, although none of those were correlated with our most significant risk SNPs (Online Resource 7). The strongest eQTL associations were observed in the breast cancer tissue dataset BC241 for the *SLC35F2* gene (rs181187590, *p* = 1.4 × 10^−5^, *r* ^2^ = 0.08, i.e., 8 % of the variation in *SLC35F2* expression was attributable to this SNP). Other eQTLs observed in this dataset included *ELMOD1* (rs181187590, *p* = 1.3 × 10^−4^, *r* ^2^ = 0.06), *EXPH5* (rs181187590, *p* = 3 × 10^−4^, *r* ^2^ = 0.054), and *ATM* (rs4987915, *p* = 3.7 × 10^−4^, *r* ^2^ = 0.05). In *The Cancer Genome Atlas* (TCGA) BC765 breast cancer dataset, the strongest associations with gene expression were observed for the non-coding RNA lLOC643923 (rs183293362, *p* = 2.3 × 10^−4^, *r* ^2^ = 0.02), *ATM* (rs4987924, *p* = 8.3 × 10^−4^, *r* ^2^ = 0.015), and *KDELC2* (rs4753834, *p* = 8.6 × 10^−4^, *r* ^2^ = 0.015) loci. The eQTL analysis performed for the TCGA normal breast tissue dataset (NB93) showed an association between SNP chr11:108075271:D and *ACAT1* gene expression level (*p* = 6.5 × 10^−3^, *r* ^2^ = 0.08). No association was observed in the normal breast tissue dataset NB116.

### Functional annotation

**Fig. 2:**
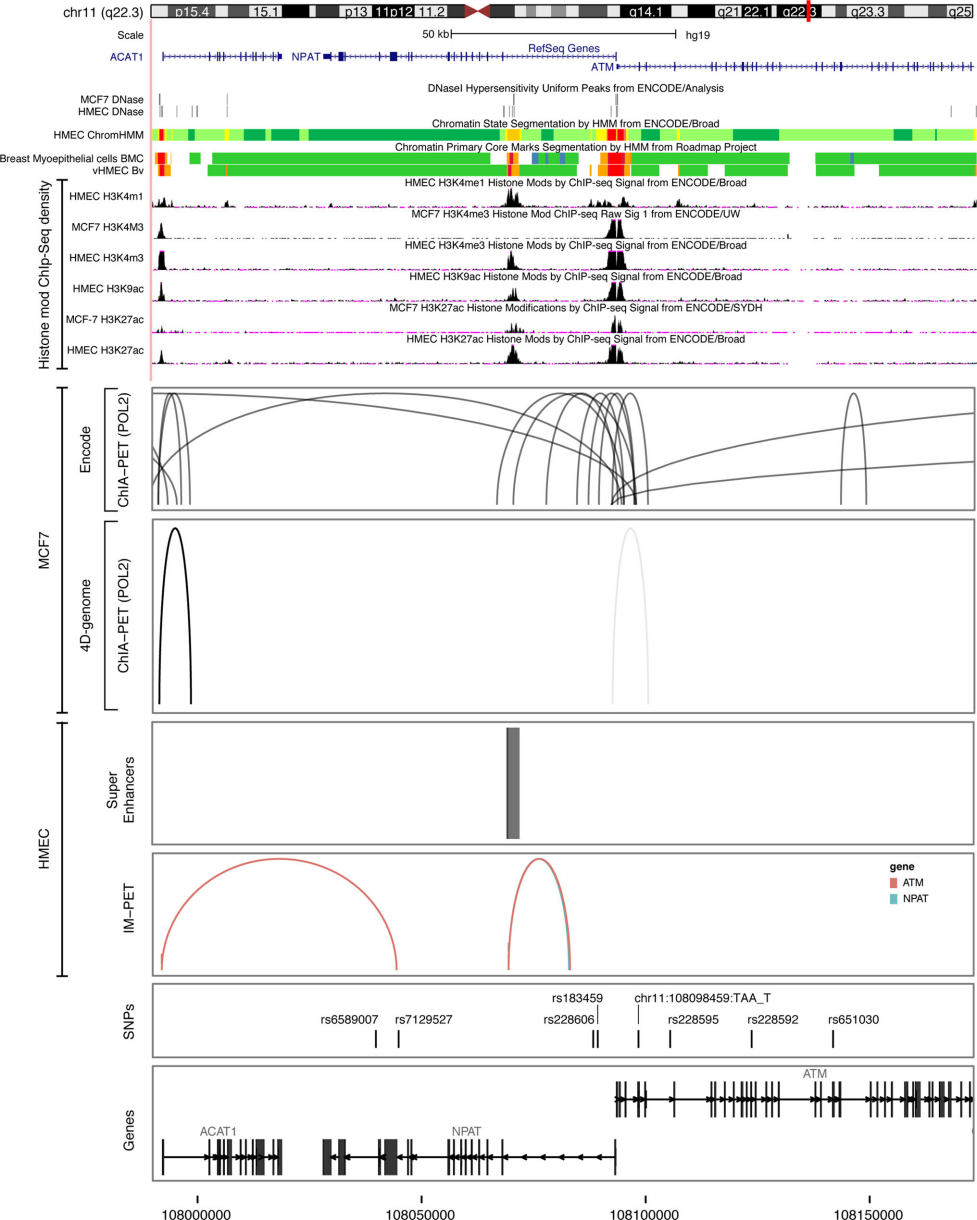
Functional annotation of the 11q22.3 locus. *Upper panel* functional annotations using data from the ENCODE and NIH Roadmap Epigenomics projects. From *top* to *bottom*, epigenetic signals evaluated included DNase clusters in MCF7 cells and HMECs, chromatin state segmentation by hidden Markov model (ChromHMM) in HMECs, breast myoepithelial cells, and variant human mammary epithelial cells (vHMECs), where *red* represents an active promoter region, *orange* a strong enhancer, and *yellow* a poised enhancer (the detailed *color scheme* of chromatin states is described in the UCSC browser), and histone modifications in MCF7 and HMEC cell lines. All tracks were generated by the UCSC genome browser (hg 19 release). *Lower panel* long-range chromatin interactions: from *top* to *bottom*, ChiA-PET interactions for RNA polymerase II in MCF-7 cells identified through ENCODE and 4D-genome. The ChiA-PET raw data available from the GEO database under the following accession (GSE33664, GSE39495) were processed with the GenomicRanges package. Maps of mammary cell super-enhancer locations as defined in Hnisz et al. [24] are shown in HMECs. Predicted enhancer–promoter determined interactions in HMECs, as defined by the integrated method for predicting enhancer targets (IM-PET), are shown. The annotation was obtained through the Bioconductor annotation package TxDb.Hsapiens.UCSC.hg19.knownGene. The tracks have been generated using ggplot2 and ggbio library in R

In order to assess the potential functional role of the most significant risk SNPs in the 11q22.3 region, ENCODE chromatin biological features were evaluated in available breast cells, namely HMECs, breast myoepithelial cells, and MCF7 breast cancer cells. We observed some overlap between features of interest and candidate SNPs within the 11q22.3 region (Fig. 2). The most interesting variant was rs228606, which overlapped a monomethylated H3K4 mark in HMECs. Analysis of data from the Roadmap Epigenomics project also showed overlap with a monomethylated H3K4 mark and with an acetylated H3K9 mark in primary breast myoepithelial cells. From ChiA-PET data, chromosomal interactions were found in the *NPAT* and *ATM* genes in MCF7 cells, located mainly in the vicinity of the promoter regions of these genes, which encompassed a strongly associated imputed SNP at this locus, namely chr11:108098459_TAA_T. Lastly, although super-enhancers and predicted enhancer–promoter interactions mapped to the 11q22.3 locus in HMECs, none overlapped with our top candidate SNPs (Fig. 2).

## Discussion

DAE is a common phenomenon in human genes, which represents a new approach to identifying *cis*-acting mechanisms of gene regulation. It offers a new avenue for the study of GWAS variants significantly associated with various diseases/traits. Indeed, the majority of GWAS hits localize outside known protein-coding regions [11, 12], suggesting a regulatory role for these variants. In the present study, we have assessed the association between 320 SNPs associated with DAE and breast/ovarian cancer risk among *BRCA1* and *BRCA2* mutation carriers. Using this approach, we found evidence of association for a region at 11q22.3, with breast cancer risk in *BRCA1* mutation carriers. Analysis of imputed SNPs across a 185-kb region (±50 kb from the center of each of the three genotyped SNPs at this locus) revealed a set of five strongly correlated SNPs that were significantly associated with breast cancer risk. This region contains several genes including *ACAT1*, *NPAT*, and *ATM*. *ACAT1* (acetyl-CoA acetyltransferase 1) encodes a mitochondrial enzyme that catalyzes the reversible formation of acetoacetyl-CoA from two molecules of acetyl-CoA. Defects in this gene are associated with ketothiolase deficiency, an inborn error of isoleucine catabolism [29]. *NPAT* (nuclear protein, co-activator of histone transcription) is required for progression through the G1 and S phases of the cell cycle, for S phase entry [30], and for the activation of the transcription of histones H2A, H2B, H3, and H4 [31]. *NPAT* germline mutations have been associated with Hodgkin lymphoma [32]. Finally, *ATM* (ataxia telangiectasia mutated) encodes an important cell cycle checkpoint kinase that is required for cellular response to DNA damage and for genome stability. Mutations in this gene are associated with ataxia telangiectasia, an autosomal recessive disorder [33]. *ATM* is also an intermediate-risk breast cancer susceptibility gene, with rare heterozygous variants being associated with increased risk of developing the disease [34]. Although several studies have assessed the role of the most common *ATM* variants in breast cancer susceptibility, the results obtained are inconsistent [35]. A recent study had identified an association between an *ATM* haplotype and breast cancer risk in *BRCA1* mutation carriers with a false discovery rate-adjusted *p* value of 0.029 for overall association of the haplotype [36]. Four of the five SNPs making up the haplotype were almost perfectly correlated (*r* ^2^ >0.9) with the three originally genotyped SNPs of the present study. These SNPs were, however, only moderately correlated (*r* ^2^ >0.4) with the most significant risk SNPs (*p* = 10^−6^), identified later through imputation.

Although eQTL analysis has identified *cis*-eQTL associations between several variants and *ACAT1*, *ATM* as well as other neighboring genes in both breast carcinoma and normal breast tissues, none of these associations involved the most significantly associated risk SNPs. Furthermore, the correlation between eQTLs and the most significant risk SNPs was weak. The lack of consistency between the eQTL results and the allelic imbalance data originally used for SNP selection in the design of the present study can probably be explained by the differences between the cell types used in these analyses. The list of allelic imbalance-associated SNPs was selected from studies performed in lymphoblastoid cell lines [15], primary skin fibroblasts [13, 16], and primary monocytes [17], while eQTLs were analyzed in breast carcinoma and normal breast tissue. This tissue heterogeneity in the data sources used represents one of the limitations of this study, although no such data were available in mammary cells when this study was originally designed.

The identification of a region at 11q22.3 that is associated specifically with breast cancer risk in *BRCA1* mutation carriers may explain why association studies performed using breast cancer cases from the general population have so far yielded conflicting results with regard to common variants at this locus. The majority of tumors arising in *BRCA1* carriers show either low or absent ER expression, while the majority of *BRCA2*-associated tumors are ER positive, as in most sporadic cancers arising in the general population. Large-scale studies using only ER-negative or triple-negative (i.e., ER-, progesterone receptor-, and HER2-negative) cases could therefore be helpful to confirm the association of this locus with breast cancer risk.

## Electronic supplementary material

Below is the link to the electronic supplementary material.

Supplementary material 1 (PDF 56 kb)

Supplementary material 2 (DOC 81 kb)

Supplementary material 3 (PDF 92 kb)

Supplementary material 4 (DOC 34 kb)

Supplementary material 5 (XLSX 281 kb)

Supplementary material 6 (XLSX 383 kb)

Supplementary material 7 (XLSX 180 kb)
