# Supplementary material for: Association of breast cancer risk in BRCA1 and BRCA2 mutation carriers with genetic variants showing differential allelic expression: identification of a modifier of breast cancer risk at locus 11q22.3
Source: Breast Cancer Res Treat. 2016 Oct 28;161(1):117–34. doi: 10.1007/s10549-016-4018-2 (PMC5222911; doi:10.1007/s10549-016-4018-2)
Supplement: Supplementary file 1 — Supplementary material 1 (PDF 56 kb) [file 10549_2016_4018_MOESM1_ESM.pdf]

Online Resource 1. Number of *BRCA1* and *BRCA2* mutation carriers by study

Acronym	Study	Country	Number of carriers
BCFR	Breast Cancer Family Registry	USA/Australia/Canada	812
BFBOCC	Baltic Familial Breast Ovarian Cancer Consortium	Latvia/Lithuania	146
BIDMC	Beth Israel Deaconess Medical Center	USA	54
BMBSA	BRCA-gene mutations and breast cancer in South African women	South Africa	207
BRICOH	Beckman Research Institute of the City of Hope	USA	304
CBCS	Rigshospitalet	Denmark	391
CNIO	Spanish National Cancer Centre	Spain	340
COH	City of Hope Cancer Center	USA	332
CONSIT TEAM	CONsorzio Studi ITaliani sui Tumori Ereditari Alla Mammella	Italy	1,323
DEMOKRITOS	National Centre for Scientific Research Demokritos	Greece	195
DFCI	Dana Farber Cancer Institute	USA	290
DKFZ	German Cancer Research Center	Germany/Pakistan/Colombia	85
EMBRACE	Epidemiological Study of Familial Breast Cancer	UK	2,479
FCCC	Fox Chase Cancer Center	USA	169
G-FAST	Ghent University Hospital	Belgium	270
GC-HBOC	German Familial Breast Group	Germany	1,516
GEMO	Genetic Modifiers of cancer risk in BRCA1/2 mutation carriers	France/USA	2,116
GEORGETOWN	Georgetown University	USA	16
GOG	Gynecologic Oncology Group	USA	657
HCSC	Hospital Clinico San Carlos	Spain	244
HEBCS	Helsinki Breast Cancer Study	Finland	218
HEBON	Genen Omgeving studie van de werkgroep Hereditiair Borstkanker Onderzoek Nederland	Netherlands	2,008
HUNBOCS	Molecular Genetic Studies of Breast- and Ovarian Cancer in Hungary	Hungary	143
HVH	University Hospital Vall d'Hebron	Spain	117
ICO	Institut Català d'Oncologia	Spain	438
IHCC	International Hereditary Cancer Centre	Poland	703
ILUH	Iceland Landspitali - University Hospital	Iceland	129
INHERIT	INterdisciplinary HEalth Research Internal Team BReast CAncer susceptibility	Canada (Quebec)	145
IOVHBOCS	Istituto Oncologico Veneto	Italy	260
IPOBCS	Portuguese Oncology Institute-Porto Breast Cancer Study	Portugal	95
KCONFAB	Kathleen Cuningham Consortium for Research into Familial Breast Cancer	Australia/New Zealand	1,167
MAGIC	Modifiers and Genetics in Cancer	USA	54
MCGILL	McGill University	Canada (Quebec)	81
MAYO	Mayo Clinic	USA	417
MOD SQUAD	Modifier Study of Quantitative Effects on Disease	Czech Republic/Belgium	174
MSKCC	Memorial Sloane Kettering Cancer Center	USA	556
MUV	General Hospital Vienna	Austria	646
NCI	National Cancer Institute	USA	241
NICCC	National Israeli Cancer Control Center	Israel	169
NNPIO	N.N. Petrov Institute of Oncology	Russia	51
OCGN	Ontario Cancer Genetics Network	Canada	391
OSU CCG	The Ohio State University Comprehensive Cancer Center	USA	148
OUH	Odense University Hospital	Denmark	573
PBCS	Università di Pisa	Italy	80
SMC	Sheba Medical Centre	Israel	367
SWE-BRCA	Swedish Breast Cancer Study	Sweden	540
UCHICAGO	University of Chicago	USA	97
UCLA	University of California Los Angeles	USA	41
UCSF	University of California San Francisco	USA	130
UKGRFOCR	UK and Gilda Radner Familial Ovarian Cancer Registries	UK/USA	77
UPENN	University of Pennsylvania	USA	655
UTMDACC	University of Texas MD Anderson Cancer Center	USA	115
VFCTG	Victorian Familial Cancer Trials Group	Australia	59
WCP	Women's Cancer Program at Cedars-Sinai Medical Center	USA	286
		**TOTAL**	**23,463**
