# Supplementary material for: Association of breast cancer risk in BRCA1 and BRCA2 mutation carriers with genetic variants showing differential allelic expression: identification of a modifier of breast cancer risk at locus 11q22.3
Source: Breast Cancer Res Treat. 2016 Oct 28;161(1):117–34. doi: 10.1007/s10549-016-4018-2 (PMC5222911; doi:10.1007/s10549-016-4018-2)
Supplement: Supplementary file 2 — Supplementary material 2 (DOC 81 kb) [file 10549_2016_4018_MOESM2_ESM.doc]

**Online Resource 2:** List of local ethics committees that granted approval for the access and use of the data in current study

| **Study** | **Country** | **Committee approval** |
| --- | --- | --- |
| Breast Cancer Family Registry (BCFR) | USA | Institutional Review Board University of Utah |
| (BCFR - additional) | Australia | The University of Melbourne Health Sciences Human Ethics Sub-Committee |
| (BCFR - additional) | USA | Columbia University Medical Center Institutional Review Board |
| (BCFR - additional) | USA | Northern California Cancer Center Institutional Review Board |
| (BCFR - additional) | Canada | University Health Network Research Ethics Board |
| (BCFR - additional) | Canada | Mount Sinai Hospital Research Ethics Board |
| Baltic Familial Breast and Ovarian Cancer Consortium (BFBOCC) | Latvia,  Lithuania | Centrālā medicīnas ētikas Komiteja |
| Beth Israel Deaconess Medical Center (BIDMC) | USA | Dana-Farber/Harvard Cancer Center Institutional Review Board |
| BRCA-gene mutations and breast cancer in South African women (BMBSA) | South Africa | Univ. of Pretoria and Pretoria Academic Hospitals Ethics Committee |
| Beckman Research Institute of the City of Hope (BRICOH) | USA | City of Hope Institutional Review Board |
| Copenhagen Breast Cancer Study (CBCS) | Denmark | De Videnskabsetiske Komiteer I Region Hovedsladen |
| Spanish National Cancer Centre (CNIO) | Spain | Instituto de Salud Carlos III Comité de Bioética y Bienestar Animal |
| City of Hope Cancer Center (COH) | USA | City of Hope Institutional Review Board |
| CONsorzio Studi ITaliani sui Tumori Ereditari Alla Mammella (CONSIT TEAM) | Italy | Comitato Etico Indipendente della Fondazione IRCCS "Istituto Nazionale dei Tumori" |
| National Centre for Scientific Research Demokritos (DEMOKRITOS) | Greece | Bioethics committee of NCSR ‘‘Demokritos’’ |
| Dana Farber Cancer Institute (DFCI) | USA | Dana-Farber/Harvard Cancer Center Institutional Review Board |
| Deutsches Krebsforschungszentrum (DKFZ) | Germany | Ethik-Kommission des Klinikums der Universität Heidelberg |
| (DKFZ - additional) | Colombia | Hospital Universitario de San Ignacio Comité de Investigaciones y Etica |
| (DKFZ - additional) | Pakistan | Shaukat Khanum Memorial Cancer Hospital and Research Centre Institutional Review Board |
| Epidemiological study of BRCA1 and BRCA2 mutation carriers (EMBRACE) | UK and EIRE | Anglia & Oxford MREC |
| Fox Chase Cancer Center (FCCC) | USA | Institutional Review Board Fox Chase Cancer Center |
| Ghent University Hospital (G-FAST) | Belgium | Universitair Ziekenhuis Gent - commissie voor medische ethiek |
| German Consortium of Hereditary Breast and Ovarian Cancer (GC-HBOC) | Germany | Ethik-Kommission der Medizinischen Fakultät der Universät zu Köln |
| Genetic Modifiers of cancer risk in *BRCA1*/2 mutation carriers (GEMO) | France | Comité consultatif sur le traitement de I'information en matière de recherche dans le domaine de la santé |
| Georgetown University (GEORGETOWN) | USA | MedStar Research Institute - Georgetown University Oncology Institutional Review Board |
| Gynecologic Oncology Group (GOG) | USA | National Cancer Institute - Cancer Prevention and Control Concept Review Committee |
| Hospital Clinico San Carlos (HCSC) | Spain | Comité Ético de Investigación Clínia Hospital Clínico San Carlos |
| Helsinki Breast Cancer Study (HEBCS) | Finland | Helsingin ja uudenmaan sairaanhoitopiiri (Helsinki University Central Hospital ethics committee) |
| HEreditary Breast and Ovarian study Netherlands (HEBON) | Netherlands | Protocol Toetsingscommissie van het Nederlands Kanker Instituut/Antoni van Leeuwenhoek Ziekenhuis |
| Hungarian Breast and Ovarian Cancer Study (HUNBOCS) | Hungary | Institutional Review Board of the Hungarian National Institute of Oncology |
| University Hospital Vall d'Hebron (HVH) | Spain | The Hospital Universitario Vall d'Hebron Clinical Research Ethics Committee |
| Institut Català d'Oncologia (ICO) | Spain | Catalan Institute of Oncology Institutional Review Board |
| International Hereditary Cancer Centre (IHCC) | Poland | Komisji Bioetycznej Pomorskiej Akademii Medycznej (Pomeranian Medical University Bioethics Committee) |
| Iceland Landspitali - University Hospital (ILUH) | Iceland | Vísindasiđanefnd National Boethics Committee |
| Interdisciplinary Health Research  International Team Breast Cancer  Susceptibility (INHERIT) | Canada | Comité d'éthique de la recherche du Centre Hospitalier Universitaire de Québec |
| Istituto Oncologico Veneto Hereditary Breast and Ovarian Cancer Study (IOVHBOCS) | Italy | Centro Oncologico Regionale Azienda Ospedale Di Padova Comitato Etico |
| Portuguese Oncology Institute-Porto Breast Cancer Study (IPOBCS) | Portugal | Comissão de Ética para a Saúde (CES) do IPO-Porto |
| Kathleen Cuningham Foundation Consortium for Research into Familial Breast Cancer (KCONFAB) | Australia | Peter MacCallum Cancer Centre Ethics Committee |
| (KCONFAB - additional) | Australia | Queensland Institute of Medical Research - Human Research Ethics Committee |
| Modifiers and Genetics in Cancer (MAGIC) | USA | University of Pennsylvania Institutional Review Board |
| Mayo Clinic (MAYO) | USA | Mayo Clinic Institutional Review Boards |
| Modifier Study of Quantitative Effects on Disease  (MOD Squad) | USA | Mayo Clinic Institutional Review Boards |
| McGill University (MCGILL) | Canada | McGill Faculty of Medicine Institutional Review Board |
| Memorial Sloane Kettering Cancer Center (MSKCC) | USA | Memorial Sloan-Kettering Cancer Center IRB |
| (MSKCC - additional) | USA | Human Biospecimen Utilization Committee |
| General Hospital Vienna (MUV) | Austria | Ethikkommission der Medizinischen Universität Wien |
| National Cancer Institute (NCI) | USA | NIH Ethics Office |
| National Israeli Cancer Control Center (NICCC) | Israel | Carmel Medical Center Institutional Review Board (Helsinki Committee) |
| N.N. Petrov Institute of Oncology (NNPIO) | Russia | N.N. Petrov Institutional Ethical Committee |
| Ontario Cancer Genetics Network (OCGN) | Canada | Mount Sinai Hospital Research Ethics Board |
| The Ohio State University Comprehensive Cancer Centre (OSU CCG) | USA | Cancer Institutional Review Board |
| Odense University Hospital (OUH) | Denmark | Den Videnskabsetiske Komité for Region Syddanmark |
| Pisa Breast Cancer Study (PBCS) | Italy | Comitato Etico per lo studio del farmaco sull'uomo (Ethics Committee for the study of the drug on humans) |
| SMC | Israel | Sheba Medical Center Institutional Review Board |
| Swedish Breast Cancer Study (SWE-BRCA) | Sweden | Regionala Etikprövningsnämnden Stockholm |
| University of Chicago (UCHICAGO) | USA | University of Chicago Biological Sciences Division IRB |
| UCLA | USA | UCLA Institutional Review Board |
| University of California San Francisco (UCSF) | USA | Committee on Human Research |
| UK and Gilda Radner Familial Ovarian Cancer Registries (UKGRFOCR) | UK | Cambridge Local Research Ethics Committee |
| (UKGRFOCR - additional) | USA | Roswell Park Cancer Institute IRB |
| University of Pennsylvania (UPENN) | USA | University of Pennsylvania Institutional Review Board |
| UTMDACC | USA | University of Texas MD Anderson Cancer Center Institutional Review Board |
| Victorian Familial Cancer Trials Group (VFCTG) | Australia | Peter MacCallum Cancer Centre Ethics Committee |
| Women’s Cancer Research Institute (WCP) | USA | Cedars-Sinai Institutional Review Board |
