# Supplementary material for: Association of breast cancer risk in BRCA1 and BRCA2 mutation carriers with genetic variants showing differential allelic expression: identification of a modifier of breast cancer risk at locus 11q22.3
Source: Breast Cancer Res Treat. 2016 Oct 28;161(1):117–34. doi: 10.1007/s10549-016-4018-2 (PMC5222911; doi:10.1007/s10549-016-4018-2)
Supplement: Supplementary file 3 — Supplementary material 3 (PDF 92 kb) [file 10549_2016_4018_MOESM3_ESM.pdf]

Online Resource 3: List of selected genes and genetic variants associated with differential allelic expression

Submitted SNP	Surrogates genotyped on the iCOGS array in replacement of original candidate SNPs	Chromosome	Position of genotyped SNPs[a]	Genes in which genotyped SNPs are located	Genes in which differential allelic expression is observed
rs11102709	rs11102707	1	114493583	*HIPK1*	*DCLRE1B*
rs1805415	rs1136410	1	226555302	*PARP1*	*PARP1*
rs11810440	rs11806633	1	227308416	*CDC42BPA*	*CDC42BPA*
rs11811771	rs11811771	1	114463049	Intergenic	*DCLRE1B*
rs12025623	rs12025623	1	21892479	*ALPL*	*ALPL*
rs1395472	rs12058802	1	91923254	Intergenic	*CDC7*
rs12087525	rs12087525	1	114489754	*HIPK1*	*DCLRE1B*
rs12093351	rs12093351	1	114529464	Intergenic	*DCLRE1B*
rs12125947	rs12125947	1	91990487	*CDC7*	*CDC7*
rs13374264	rs13374264	1	46683014	*LURAP1 / POMGNT1*	*RAD54L*
rs13447450	rs13447450	1	91965850	*CDC7*	*CDC7*
rs13447454	rs13447454	1	91966406	*CDC7*	*CDC7*
rs1767429	rs1767429	1	21889340	*ALPL*	*ALPL*
rs2020902	rs2020902	1	15834360	*CASP9*	*CASP9*
rs11119792	rs2028706	1	211831380	*NEK2*	*NEK2*
rs2233025	rs2233025	1	11735652	*MAD2L2*	*MAD2L2*
rs2336030	rs2336030	1	11736600	*MAD2L2*	*MAD2L2*
rs2805053	rs2805053	1	162781787	*HSD17B7*	*HSD17B7*
rs2911571	rs2911571	1	85156821	*SSX2IP*	*PRKACB*
rs6667862	rs1052576	1	15832543	*CASP9*	*CASP9*
rs4645989	rs4645989	1	15850343	*CASP9*	*CASP9*
rs4646047	rs4646047	1	15831783	*CASP9*	*CASP9*
rs4646091	rs4646091	1	15822043	*CASP9*	*CASP9*
rs471144	rs471144	1	152454255	Intergenic	*MCl-1*
rs513826	rs513826	1	38166022	*CDCA8*	*CDCA8*
rs17485896	rs522712	1	91943207	Intergenic	*CDC7*
rs6668665	rs6673329	1	162794500	Intergenic	*HSD17B7*
rs6674384	rs6674384	1	114455907	*DCLRE1B*	*DCLRE1B*
rs6676779	rs6676779	1	243875486	*AKT3*	*AKT3*
rs510208	rs686083	1	38149193	*C1orf109*	*CDCA8*
rs697004	rs697004	1	211842808	*NEK2*	*NEK2*
rs7521639	rs7521639	1	227223736	*CDC42BPA*	*CDC42BPA*
rs7546172	rs7546172	1	227622011	*CDC42BPA*	*CDC42BPA*
rs885814	rs885814	1	21875916	*ALPL*	*ALPL*
rs9793263	rs9793263	1	46722389	*RAD54L*	*RAD54L*
rs4675093	rs10205923	2	227614557	*IRS1*	*IRS1*
rs10490250	rs10490250	2	58509628	Intergenic	*FANCL*
rs10495944	rs10495944	2	47703290	*MSH2*	*MSH2*
rs11674814	rs11674814	2	202153846	*ALS2CR12*	*STRADB*
rs11903456	rs11903456	2	58393836	*FANCL*	*FANCL*
rs12464787	rs12464787	2	179440029	*TTN-AS1/TTN*	*TTN*
rs748869	rs12477063	2	215673664	*BARD1/LOC101928103*	*BARD1*
rs13026390	rs13001462	2	215678346	*LOC101928103*	*BARD1*
rs13019654	rs13019654	2	47689217	*MSH2*	*MSH2*
rs13021937	rs13021937	2	215673937	*BARD1*	*BARD1*
rs1421	rs1421	2	47613870	*EPCAM*	*MSH2*
rs16822638	rs16822647	2	227651123	*IRS1*	*IRS1*
rs11890159	rs17702263	2	173459590	*PDK1*	*PDK1*
rs17758988	rs2063018	2	28318008	*BRE*	*BRE*
rs207928	rs207928	2	217036441	*XRCC5*	*XRCC5*
rs2305354	rs2305354	2	100019496	*REV1*	*REV1*
rs4075604	rs4075604	2	173387668	Intergenic	*PDK1*
rs4233638	rs4663992	2	234889177	*TRPM8*	*UGT1A1*
rs668844	rs668844	2	217021602	*XRCC5*	*XRCC5*
rs6715570	rs6715570	2	215673440	*BARD1*	*BARD1*
rs6721310	rs6721310	2	28319320	*BRE*	*BRE*
rs6757091	rs6757091	2	215672793	*BARD1*	*BARD1*
rs726379	rs726379	2	231685198	*CAB39*	*CAB39*
rs7609252	rs12052479	2	173404804	*BUB1*	*BUB1*
rs768298	rs768298	2	58410972	*FANCL*	*FANCL*
rs6443276	rs13066757	3	10045030	*EMC3-AS1*	*FANCD2*
rs13073141	rs13073141	3	51410485	*DOCK3*	*rad54L2*
rs13082648	rs13082648	3	142129008	*XRN1*	*ATR*
rs13099560	rs13099560	3	48204768	*CDC25A*	*CDC25A*
rs16838754	rs16838754	3	131843104	Intergenic	*TOPBP1*
rs2087760	rs2087760	3	27375730	*SLC4A7*	*SLC4A7*
rs1043943	rs4103004	3	20042834	*PP2D1*	*XPC*
rs4241370	rs4241370	3	133794199	Intergenic	*RYK*
rs10804625	rs4552343	3	133842112	Intergenic	*RYK*
rs6807775	rs6807775	3	65133697	Intergenic	*BAP1*
rs7611218	rs7611218	3	10063960	*CIDECP*	*FANCD2*
rs7626117	rs7626117	3	10140671	*FAND2*	*FANCD2*
rs6795164	rs7639683	3	133973731	Intergenic	*RYK*
rs7653368	rs7653368	3	47985361	*CDC25A*	*CDC25A*
rs803335	rs803335	3	10085035	*FANCD2*	*FANCD2*
rs4693089	rs11099601	4	84382763	*FAM175A*	*FAM175A*
rs2866416	rs11946020	4	103944742	*SLC9B2*	*UBE2D3*
rs1354360	rs1354360	4	70616322	*SULT1B1*	*SULT1E1*
rs1389999	rs1389999	4	14858341	*LINC00504*	*CPEB2*
rs1503721	rs1503721	4	70679571	Intergenic	*SULT1E1*
rs223385	rs150895	4	103712589	*4:102791432*	*UBE2D3*
rs17355027	rs17355027	4	84388915	*FAM175A*	*FAM175A*
rs17802877	rs17802877	4	1720346	*TMEM129*	*TACC3*
rs223497	rs223497	4	103653976	*MANBA*	*UBE2D3*
rs93059	rs230530	4	103453980	*NFKB1*	*UBE2D3*
rs11722394	rs2788865	4	26446006	Intergenic	*RB1*
rs2854915	rs2854915	4	1721654	*TACC3/TMEM129*	*TACC3*
rs3774936	rs3774937	4	103434253	*NFKB1*	*NFKB*

rs4647601	rs4647601	4	185570032	*CASP3*	*CASP3*
rs4647616	rs4647610	4	185567931	*CASP3*	*CASP3*
rs6830624	rs6830624	4	104020422	*BDH2*	*CENPE*
rs11728699	rs7439493	4	110656730	Intergenic	*CASP6*
rs798755	rs798755	4	1720824	*TMEM129*	*TACC3*
rs1494961	rs971539	4	84433804	Intergenic	*FAM175A*
rs2168805	rs980455	4	103418957	Intergenic	*NFKB*
rs9998559	rs9998559	4	15113921	Intergenic	*CPEB2*
rs12517451	rs12517451	5	79920073	Intergenic	*MSH3*
rs878234	rs13171253	5	43160433	*ZNF131*	*MGC42105/ NIM1K*
rs13176284	rs13176284	5	34754248	*RAI14*	*Rad1*
rs2915794	rs1432693	5	147609136	Intergenic	*SPINK7*
rs1650697	rs1650697	5	79950781	*DHFR et MSH3*	*MSH3*
rs17772583	rs17772583	5	131953510	*Rad50*	*Rad50*
rs2362974	rs2362974	5	36156654	*SKP2*	*SKP2*
rs374745	rs2546163	5	76995054	*TBCA*	*TBCA*
rs2652218	rs2652217	5	76980244	Intergenic	*TBCA*
rs2662370	rs2662370	5	76981357	Intergenic	*TBCA*
rs2706369	rs2706369	5	131932203	*Rad50*	*Rad50*
rs29636	rs29635	5	171024355	Intergenic	*NPM1*
rs359454	rs359445	5	173285204	Intergenic	*CPEB4*
rs3797306	rs3797306	5	43127925	*ZNF131*	*MGC42105*
rs425463	rs425463	5	79901952	Intergenic	*MSH3*
rs4302608	rs4302608	5	170840504	Intergenic	*NPM1*
rs6151599	rs6151599	5	79951032	*DHRF et MSH3*	*MSH3*
rs6453340	rs6453340	5	76962274	Intergenic	*TBCA*
rs7702272	rs7702272	5	36160854	*SKP2*	*SKP2*
rs7707008	rs7707008	5	170801952	Intergenic	*NPM1*
rs7722173	rs7722173	5	176384683	*UIMC1*	*UIMC1*
rs1077394	rs1077394	6	31610384	*BAG6*	*AIF*
rs1144708	rs1144708	6	31710020	*MSH5-SAPCD1*	*AIF*
rs11753187	rs11753187	6	111781972	*REV3L*	*REV3*
rs12208908	rs12208908	6	109048366	Intergenic	*FOXO3*
rs1159806	rs1268166	6	109008232	Intergenic	*FOXO3*
rs13210247	rs13210247	6	111922720	*TRAF3IP2*	*FYN*
rs4947349	rs1480380	6	32913246	Intergenic	*BRD2*
rs17254634	rs17254634	6	80751942	*TTK*	*TTK*
rs195385	rs195385	6	37333796	*RNF8*	*RNF8*
rs206776	rs206776	6	32953711	Intergenic	*BRD2*
rs210170	rs210170	6	33525680	Intergenic	*BAK1*
rs2179070	rs2179070	6	111885752	*TRAF3IP2-AS1*	*REV3L*
rs2269058	rs2269058	6	37324857	*RNF8*	*RNF8*
rs2280152	rs2280152	6	112375889	*WISP3*	*FYN*
rs2284923	rs2284923	6	37348920	*RNF8*	*RNF8*
rs3106191	rs3106191	6	33280505	*TAPBP*	*DAXX*
rs3130000	rs3130000	6	30628082	*DHX16*	*MDC1*
rs3176326	rs3176326	6	36647289	*CDKN1A*	*CDKN1A*
rs3777947	rs3777947	6	112433664	Intergenic	*FYN*
rs396746	rs396746	6	33557045	*GGNBP1*	*BAK1*
rs465969	rs465969	6	111655530	*REV3L*	*REV3L*
rs4710127	rs4710127	6	167276041	*RPS6KA2*	*RPS6KA2*
rs671271	rs671271	6	112261385	Intergenic	*FYN*
rs726230	rs726230	6	11200574	*NEDD9*	*NEDD9*
rs733590	rs733590	6	36645203	*CDKN1A*	*CDKN1A*
rs1740377	rs809193	6	112020414	*FYN*	*FYN*
rs9276931	rs9276931	6	32928984	Intergenic	*BRD2*
rs9296095	rs9296095	6	33542523	*BAK1*	*BAK1*
rs9386747	rs9386747	6	109038738	Intergenic	*FOXO3*
rs9457245	rs9457245	6	167340715	Intergenic	*RPS6KA2*
rs9468322	rs9468322	6	28231243	Intergenic	*NKAPL*
rs1534078	rs10241838	7	96343665	Intergenic	*SHFM1*
rs12666800	rs12666800	7	45906594	Intergenic	*IGFBP-1*
rs6968090	rs6968090	7	96318575	*SHFM1*	*SHFM1*
rs6948097	rs7804722	7	92288106	*CDK6*	*CDK6*
rs10104637	rs10104637	8	95393925	*RAD54B*	*RAD54B*
rs1011683	rs1011683	8	91020834	*DECR1*	*NBN*
rs11574158	rs11574158	8	30891353	*PURG/WRN*	*WRN*
rs11574398	rs11574398	8	31025343	*WRN*	*WRN*
rs11786591	rs11786591	8	74916344	*8q21*	*8q21*
rs13279327	rs13279327	8	48442384	*SPIDR*	*PRKDC*
rs17525603	rs17525603	8	80588407	Intergenic	*ESR1*
rs11779458	rs2445614	8	128212594	Intergenic	*Intergenic*
rs2515102	rs2450551	8	95351843	Intergenic	*RAD54B*
rs6982040	rs6982040	8	48708742	*PRKDC*	*PRKDC*
rs1056171	rs1056171	9	133761001	*ABL1*	*ABL1*
rs10972278	rs10972278	9	35000289	Intergenic	*FANCG*
rs11535662	rs11535662	9	140143801	*9orf173 /FAM166A*	*NELFB (COBRA1)*
rs12004745	rs12004745	9	21812349	*MTAP*	*CDKN2B*
rs4284139	rs16923815	9	100480357	Intergenic	*XPA*
rs2808682	rs2039971	9	21815166	*MTAP*	*XPA*
rs2069422	rs2069422	9	22008026	*CDKN2B*	*CDKN2B*
rs28631372	rs28631372	9	140148051	*C9orf173*	*NEFLB (COBRA1)*
rs595429	rs595429	9	35055669	*VCP*	*FANCG*
rs6597642	rs6597642	9	133725767	*ABL1*	*ABL1*
rs10829172	rs10764686	10	27475494	*MASTL*	*MASTL*
rs1127687	rs1127687	10	115490109	*CASP7*	*CASP7*
rs11574852	rs11574852	10	104161475	*NFKB2*	*NFKB2*
rs7099427	rs12255933	10	126579158	Intergenic	*BID*
rs12411618	rs12411618	10	94052164	*CPEB*	*CPEB*
rs2260184	rs1904415	10	62540453	*CDK1*	*CDK1*
rs1937881	rs1937881	10	5066511	*AKR1C3*	*AKR1C3*
rs2227309	rs2227309	10	115489167	*CASP7*	*CASP7*
rs2419851	rs2419851	10	115380608	*NRAP*	*CASP7*
rs3780926	rs3780926	10	27436295	*YME1L1*	*MASTL*

rs3814231	rs3814231	10	115481018	*CASP7*	*CASP7*
rs11191389	rs4147155	10	104536771	*WBP1L*	*CYP17A1*
rs4353229	rs4353229	10	115489589	*CASP7*	*CASP7*
rs4919438	rs6584354	10	102007714	*CWF19L1*	*CHUK*
rs7091128	rs7091128	10	27466473	*MASTL*	*MASTL*
rs788221	rs788209	10	27434716	*YME1L1*	*MASTL*
rs7900024	rs7900024	10	27444763	*MASTL*	*MASTL*
rs882474	rs882474	10	27477865	Intergenic	*MASTL*
rs11020789	rs10765682	11	94198269	*MRE11A*	*MRE11*
rs10768975	rs10838158	11	43752522	*HSD17B12*	*HSD17B12*
rs11555762	rs11555762	11	43876698	*HSD17B12*	*AR*
rs11606458	rs11606458	11	6623918	*ILK*	*ILK*
rs12271212	rs12271212	11	77197817	Intergenic	*PAK1*
rs17140162	rs17140162	11	125490616	*STT3A*	*CHEK1*
rs1805363	rs1805363	11	94226952	*MRE11*	*MRE11*
rs624366	rs183459	11	108089197	*NPAT*	*ATM*
rs2255538	rs2255538	11	6629042	*ILK*	*ILK*
rs830084	rs2279238	11	47282024	*NR1H3*	*DDB2*
rs228589	rs228592	11	108123189	*ATM*	*ATM*
rs2862961	rs2862961	11	43656535	*LOC101928704*	*AR*
rs2957873	rs2957873	11	47249294	*DDB2*	*DDB2*
rs3212907	rs3212907	11	69468776	*CCND1*	*CCND1*
rs2013867	rs326222	11	47259668	*DDB2*	*DDB2*
rs3814707	rs3814707	11	65560785	*OVOL1*	*KAT5*
rs7926512	rs4244812	11	65480768	*KAT5*	*KAT5*
rs4435030	rs4435030	11	74806005	Intergenic	*RB1*
rs4647709	rs4647709	11	47237359	*DDB2*	*DDB2*
rs601046	rs511484	11	100918433	*PGR*	*PGR*
rs656040	rs656040	11	65621057	*SNX32*	*Mus81*
rs6589007	rs6589007	11	108040104	*NPAT*	*ATM*
rs6592537	rs6592537	11	70172975	*PPFIA1*	*FADD*
rs660442	rs660442	11	64042997	*BAD*	*BAD*
rs7110437	rs7110437	11	43780553	*HSD17B12*	*HSD17B12*
rs7118659	rs7118659	11	43817125	*HSD17B12*	*HSD17B12*
rs7131110	rs7131110	11	43846850	*HSD17B12*	*HSD17B12*
rs7928422	rs7928422	11	43749199	*HSD17B12*	*HSD17B12*
rs7928685	rs7928685	11	6773439	Intergenic	*ILK*
rs7951583	rs7951583	11	9573518	*LOC105376543*	*WEE1*
rs9667035	rs9667035	11	1907653	*LSP1*	*LSP1*
rs10160981	rs10160981	12	110904075	*GPN3*	*RAD9B*
rs7301841	rs10842893	12	27422886	*STK38L*	*STK38L*
rs7313628	rs10846535	12	124130388	*GTF2H3*	*GTF2H3*
rs10876864	rs10876864	12	56401085	*IKZF4*	*CDK2*
rs4758677	rs11060333	12	122678607	*LRRC43*	*DIABLO*
rs12368451	rs12368451	12	99115661	*APAF1*	*APAF1*
rs1291351	rs1291351	12	13084082	*CDKN1B*	*CDKN1B*
rs1502337	rs1502337	12	111062852	*TCTN1*	*RAD9B*
rs17497606	rs17497606	12	27494449	*ARNTL2*	*STK38L*
rs203343	rs203343	12	120233601	*CIT*	*CIT*
rs2066827	rs2066827	12	12871099	*CDKN1B*	*CDKN1B*
rs2069502	rs2069502	12	58144665	*CDK4*	*CDK4*
rs2162679	rs2162679	12	102871259	*IGF1*	*IGF1*
rs2996028	rs2996028	12	120165155	*CIT*	*CIT*
rs3850952	rs3850952	12	124090802	*DDX55*	*GTF2H3*
rs6488889	rs6488889	12	124144793	*GTF2H3*	*GTF2H3*
rs6490269	rs6490269	12	120207175	*CIT*	*CIT*
rs841626	rs703842	12	58162739	*CYP27B1/METTL1*	*STK38L*
rs7971802	rs708170	12	27237085	*C12orf71*	*STK38L*
rs7967755	rs7967755	12	986004	*WNK1*	*Rad52*
rs7971725	rs7971725	12	98981483	Intergenic	*Apaf1*
rs904654	rs904654	12	120189888	*CIT*	*CIT*
rs4964035	rs922275	12	27402709	*STK38L*	*STK38L*
rs12870195	rs11164141	13	114259570	*TFDP1*	*TFDP1*
rs7325708	rs12870278	13	103543243	*METTL21EP*	*ERCC5*
rs1408047	rs1408047	13	103484918	*BIVM-ERCC5*	*ERCC5*
rs1887894	rs1887894	13	21607600	*lasts2*	*Lasts2*
rs2232642	rs2232642	13	108861048	*LIG4*	*LIG4*
rs4119478	rs4119478	13	73254212	Intergenic	*C13ORF34/BORA*
rs7317356	rs4150792	13	114288971	*TFDP1*	*TFDP1*
rs7335262	rs7328482	13	21877418	*LINC00539/MIPEPP3*	*LATS2*
rs4545698	rs9509492	13	21608971	*LATS2*	*TFDP1*
rs2877455	rs12880109	14	68813032	*RAD51B*	*RAD51L1*
rs17105760	rs17105760	14	68984500	*RAD51B (Rad51L1)*	*RAD51B (Rad51L1)*
rs17106237	rs17106237	14	69203145	Intergenic	*RAD51B (Rad51L1)*
rs11160756	rs2896489	14	104108743	*KLC1*	*XRCC3*
rs2357479	rs3020445	14	64788644	*ESR2*	*ESR2*
rs4983559	rs4983559	14	105277209	Intergenic	*AKT1*
rs861537	rs861537	14	104167075	*KLC1 /XRCC3*	*XRCC3*
rs11072076	rs11072076	15	69473147	*GLCE*	*SMAD3*
rs7180135	rs12592524	15	41003463	*RAD51*	*RAD51*
rs12902785	rs12902785	15	66928241	Intergenic	*SMAD3*
rs12908284	rs12908284	15	40999504	*RAD51*	*RAD51*
rs1565866	rs1565866	15	40455894	*BUB1B*	*BUB1B*
rs2118612	rs2118612	15	67400490	*SMAD3*	*SMAD3*
rs2619679	rs2619679	15	40986237	*RAD51-AS1*	*RAD51*
rs389480	rs389480	15	91351930	*BLM*	*BLM*
rs4779041	rs4779041	15	83337059	*AP3B2/LOC283692*	*CPEB1*
rs570933	rs570933	15	43824030	*MAP1A*	*TP53BP1*
rs7178117	rs6494633	15	67423306	*SMAD3*	*SMAD3*
rs7165509	rs7165509	15	40945900	*CASC5*	*Rad51*
rs7178873	rs7178873	15	40489387	*BUB1B*	*BUB1B*
rs1061621	rs1061621	16	1376892	*UBE2I*	*UBE2I*
rs11076628	rs11076628	16	89875246	*FANCA*	*FANCA*
rs11643201	rs11643201	16	83312375	*CDH13*	*HSD17B2*

rs2028265	rs12051039	16	78488305	*WWOX*	*WWOX*
rs12446804	rs12447418	16	19539858	*CCP110*	*CP110*
rs12923828	rs12923828	16	78265497	*WWOX*	*WWOX*
rs16947165	rs16947165	16	78159277	*WWOX*	*WWOX*
rs1886701	rs1886701	16	68676932	*CDH3*	*CDH1*
rs2023671	rs2023671	16	23849839	*PRKCB*	*PRKCB*
rs8052688	rs2268049	16	1371154	*UBE2I*	*UBE2I*
rs249941	rs249941	16	23644738	*PALB2*	*PALB2*
rs2745098	rs2745098	16	1536535	*PTX4*	*UBE2I*
rs3136042	rs3136042	16	14014055	*ERCC4*	*ERCC4*
rs2159113	rs4785722	16	89863110	*ZNF276*	*FANCA*
rs3785403	rs6500437	16	89789898	*ZNF276*	*FANCA*
rs7189213	rs6564520	16	78306219	*WWOX*	*WWOX*
rs8057970	rs7135	16	19514483	*GDE1*	*CP110*
rs7195066	rs7195066	16	89836323	*FANCA*	*FANCA*
rs7195624	rs7195624	16	9010213	*USP7*	*USP7*
rs7302	rs7302	16	1375263	*UBE2I*	*UBE2I*
rs8061528	rs8061528	16	3656482	*SLX4*	*BTBD12*
rs9927946	rs9927946	16	9083367	Intergenic	*USP7*
rs9935995	rs9935995	16	19592730	*C16orf62*	*CP110*
rs12952884	rs12952884	17	49226051	Intergenic	*NME1*
rs17734	rs17734	17	1801144	*RPA1*	*RPA1*
rs17761467	rs17761467	17	1805677	Intergenic	*RPA1*
rs2854152	rs17817865	17	53032425	*STRADA*	*STRADA*
rs3744768	rs2070776	17	62007498	*CD79B*	*RPA1*
rs2078486	rs2078486	17	7583083	*TP53*	*SHBG*
rs2239680	rs2239680	17	76219783	*BIRC5*	*BIRC5*
rs9891949	rs2278637	17	8062102	*VAMP2*	*AURKB*
rs1042522	rs2287498	17	7592560	*TP53/WRAP53*	*TP53*
rs3027247	rs3027247	17	8130867	*CTC1*	*AURKB*
rs1468501	rs3826392	17	11922904	*MAP2K4*	*MAP2K4*
rs4252596	rs4252596	17	37855834	*ERBB2*	*ERBB2*
rs4794136	rs4794136	17	48433958	*XYLT2*	*Eme-1*
rs7217885	rs7217885	17	48405172	Intergenic	*Eme-1*
rs8066463	rs8066463	17	61818390	*STRADA*	*STRADA*
rs8079273	rs8079273	17	7597478	*WRAP53*	*P53*
rs9911630	rs9911630	17	41188342	*APAF1:ANKS1B*	*BRCA1*
rs11082221	rs11082221	18	20531677	*RBBP8*	*RBBP8*
rs12966424	rs12966424	18	12678486	*Cep76*	*Cep76*
rs13381022	rs13381022	18	18532756	*ROCK1*	*ROCK1*
rs4800640	rs4800640	18	18969404	*GREB1L*	*ROCK1*
rs6567131	rs6567131	18	57579140	Intergenic	*PMAIP1*
rs16973592	rs7234479	18	20599564	*RBBP8*	*RBBP8*
rs9304261	rs9304261	18	20606596	*RBBP8*	*RBBP8*
rs16973631	rs9319956	18	57594068	Intergenic	*RBBP8*
rs4556910	rs16963425	19	30340724	Intergenic	*CCNE1*
rs12052070	rs17294094	19	48720926	*CARD8*	*AR*
rs1805419	rs1805419	19	49459104	*BAX*	*BAX*
rs2298881	rs2298881	19	45926916	*ERCC1*	*ERCC1*
rs3764640	rs3764640	19	1207238	*STK11*	*STK11*
rs1009316	rs3817074	19	49459212	*BAX*	*BAX*
rs7247515	rs7247515	19	40755915	*AKT2*	*AKT*
rs751731	rs751731	19	33463058	*C19ORF40*	*C19ORF40*
rs17362677	rs16979815	20	54941087	*FAM210B*	*AURKA*
rs2267857	rs2267860	20	43655135	*STK4*	*STK4*
rs2422978	rs2422978	20	4763108	*RASSF2*	*PCNA*
rs4815608	rs4813643	20	3787755	*LOC101929125*	*CDC25B*
rs6088857	rs6060446	20	30231637	*COX4I2*	*BCL2L1*
rs6086987	rs6086987	20	9685155	*PAK7*	*PAK7*
rs6133777	rs6133777	20	9995950	Intergenic	*PAK7*
rs85021	rs85021	20	45179495	*OCSTAMP*	*ube2c*
rs910671	rs910671	20	43601921	*STK4*	*STK4*
rs988166	rs988166	20	54920858	*AURKA*	*AURKA*
rs235314	rs235314	21	46271452	*PTTG1IP*	*SUMO3*
rs2838690	rs2838690	21	46222486	*LINC01424/UBE2G2*	*SUMO3*
rs9306116	rs2838693	21	46227872	*SUMO3*	*SUMO*
rs4239838	rs4239838	21	46202309	*UBE2G2*	*SUMO3*
rs104664	rs104664	22	45711854	*FAM118A*	*SMC1B*
rs138691	rs138691	22	39115310	*GTPBP1*	*RB1*
rs181390	rs181390	22	18222263	*BID*	*BID*
rs4822002	rs4822011	22	41550954	*EP300*	*EP300*
rs5762814	rs5762814	22	29203964	Intergenic	*CHEK2*
rs737866	rs737866	22	19930109	*COMT*	*COMT*
rs9620797	rs738200	22	28792887	*TTC28*	*CHEK2*
rs738207	rs738207	22	35829452	*MCM5*	*MCM5*
rs740603	rs740603	22	19945177	*COMT*	*COMT*
rs9332362	rs9332362	22	19949103	*COMT*	*COMT*
rs9605031	rs9605031	22	19921378	*TXNRD2*	*COMT*
rs9607761	rs9607761	22	41371418	Intergenic	*EP300*

Build 37 coordinates
