# Supplementary material for: Association of breast cancer risk in BRCA1 and BRCA2 mutation carriers with genetic variants showing differential allelic expression: identification of a modifier of breast cancer risk at locus 11q22.3
Source: Breast Cancer Res Treat. 2016 Oct 28;161(1):117–34. doi: 10.1007/s10549-016-4018-2 (PMC5222911; doi:10.1007/s10549-016-4018-2)
Supplement: Supplementary file 4 — Supplementary material 4 (DOC 34 kb) [file 10549_2016_4018_MOESM4_ESM.doc]

**Online Resource** 4: List of datasets used for eQTL analyses

| **Datasets** | **Description** | **Genotype data source** | **Expression data source** | **References** |
| --- | --- | --- | --- | --- |
| **Normal Breast** | | | | |
| **NB116** | Samples from women of Caucasian ancestry living in Norway, comprising expression data from normal breast biopsies (n=73), reduction plastic surgery (n=34) and adjacent normal (n=9) (adjacent to tumour). | iCOGS SNP array | Gene expression levels were measured with Agilent 44K | 1, 2 |
| **NB93** | Caucasian fraction of the TCGA BRCA dataset for which adjacent normal breast expression data were available (n=93). | Birdseed processed germline genotype data from the Affy6 SNP array were obtained from the TCGA dbGAP data portal. | Gene expression levels were assayed by RNA sequencing, RSEM (RNAseq by Expectation-Maximization) normalized per gene, as obtained from the TCGA consortium. The data was log2 transformed, and unexpressed genes were excluded prior to eQTL analysis. | 3, 4 |
| **Breast carcinomas** | | | | |
| **BC241** | Caucasian sample set recruited from Norway, n=241. The sample set includes all stages of breast cancer. | iCOGS SNP array | mRNA expression data was from the Agilent 44K array | 2, 5 |
| **BC765** | Samples from the TCGA breast cancer sample set of Caucasian origin n=765. | Birdseed processed germline genotype data from the Affy6 SNP array were obtained from the TCGA dbGAP data portal. | Gene expression levels were assayed by RNA sequencing, RSEM (RNAseq by Expectation-Maximization) normalized per gene, as obtained from the TCGA consortium. The data was log2 transformed, and unexpressed genes were excluded prior to eQTL analysis. | 3, 4 |

**References**

1. Haakensen VD, Lingjaerde OC, Lüders T, Riis M, Prat A, Troester MA, et al. Gene expression profiles of breast biopsies from healthy women identify a group with claudin-low features. BMC Med Genomics 2011;4:77.

2. Quigley DA, Fiorito E, Nord S, Van Loo P, Alnæs GG, Fleischer T, et al. The 5p12 breast cancer susceptibility locus affects MRPS30 expression in estrogen-receptor positive tumors. Mol Oncol 2014;8:273-84.

3. Cancer Genome Atlas Network. Comprehensive molecular portraits of human breast tumours. Nature 2012;490:61-70.

4. Li B, Ruotti V, Stewart RM, Thomson JA, Dewey CN. RNA-Seq gene expression estimation with read mapping uncertainty. Bioinformatics 2010;26:493-500.

5. Naume B, Zhao X, Synnestvedt M, Borgen E, Russnes HG, Lingjaerde OC, et al. Presence of bone marrow micrometastasis is associated with different recurrence risk within molecular subtypes of breast cancer. Mol Oncol 2007;1:160-71.
